# A Generalized Transformer-Based Pulse Detection Algorithm

**DOI:** 10.1021/acssensors.2c01218

**Published:** 2022-08-30

**Authors:** Dario Dematties, Chenyu Wen, Shi-Li Zhang

**Affiliations:** † Northwestern Argonne Institute of Science and Engineering, Northwestern University, 2205 Tech Drive Suite 1-160, Evanston, 60208 Illinois, United States; ‡ Mathematics and Computer Science Division, Argonne National Laboratory, 9700 S. Cass Avenue, Lemont, 60439 Illinois, United States; § NanoDynamicsLab, Laboratory of Biophysics, Wageningen University, Stippeneng 4, Wageningen 6708 WE, The Netherlands; ∥ Department of Bionanoscience, Kavli Institute of Nanoscience, Delft University of Technology, Van der Maasweg 9, Delft 2629 HZ, The Netherlands; ⊥ Department of Electrical Engineering, Uppsala University, Lägerhyddsvägen 1, 752 37, SE-751 03 Uppsala, Sweden

**Keywords:** spike recognition, machine learning, artificial neural network, transformer, nanopore sensing, generalized algorithm

## Abstract

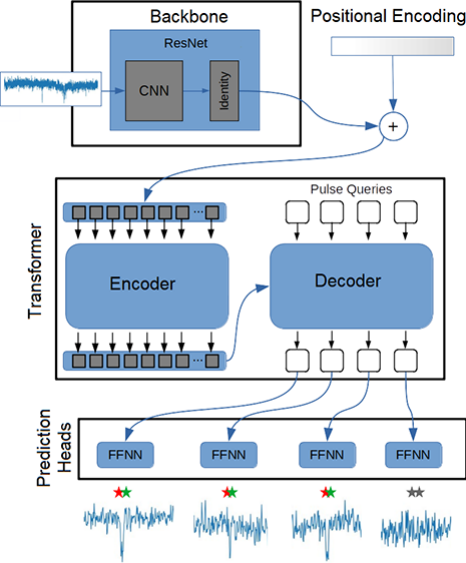

Pulse-like signals are ubiquitous in the field of single molecule analysis, *e.g.*, electrical or optical pulses caused by analyte translocations in nanopores. The primary challenge in processing pulse-like signals is to capture the pulses in noisy backgrounds, but current methods are subjectively based on a user-defined threshold for pulse recognition. Here, we propose a generalized machine-learning based method, named pulse detection transformer (PETR), for pulse detection. PETR determines the start and end time points of individual pulses, thereby singling out pulse segments in a time-sequential trace. It is objective without needing to specify any threshold. It provides a generalized interface for downstream algorithms for specific application scenarios. PETR is validated using both simulated and experimental nanopore translocation data. It returns a competitive performance in detecting pulses through assessing them with several standard metrics. Finally, the generalization nature of the PETR output is demonstrated using two representative algorithms for feature extraction.

Single-molecule analysis (SMA) technologies are developed to interrogate individual molecules so as to gain high-fidelity information about them, a task often difficult or even impossible to attain using ensemble averages.^1^ They offer a powerful toolbox for direct observation of molecule dynamics in nanoscale, *e.g.*, in peptide/protein folding,^2,3^ protein dynamics,^4,5^ single-ion electrochemical reactions,^6,7^ DNA hybridization,^8,9^*etc*. Pulse-like signals are ubiquitously found in the field of SMA. They carry comprehensive information about the concerned molecules including their dynamics and interactions with the surroundings. They can be in form of, *e.g.*, variations in electrical current through protein ion channels in cell membranes,^10^ alternations in luminance caused by analyte translocations in nanopores,^11^ and changes in electrical current related to single-molecule electrochemical reactions on nanoscale electrodes.^12^ As a typical example, nanopore sensors have been used for molecular analysis at the single-molecule level,^13^ such as DNA and protein sequencing,^14,15^ protein profiling,^16^ peptide recognition,^17^ and small molecule detection.^18^ In a nanopore, the passage of single molecules, *i.e.*, the analytes, leads to temporal blockages of the pore and, therefore, sporadic appearance of spikes on the ionic current in electrical readout or changes in luminance in optical readout. Abundant information about the translocating analytes is hidden in the fluctuating monitoring ionic current contributed from interactions between the analytes and the nanopore.^19^ Such subtle informative details in the signal are inevitably affected by noise and physical limitation of the signal readout such as the bandwidth.^20^ Hence, a prerequisite to analyze them for various purposes, such as feature extraction and classification, is to be able to single them out from the noisy background.

The commonly adopted methods to detect the pulses from time-sequential traces are based on user-defined thresholds referring to the baseline.^21−23^ Additional mechanisms are involved to self-adapt to variations or fluctuations of the baseline, typified by dynamic window average^21,24^ and iterative detection.^22^ By detection of abrupt changes in the current traces, the CUSUM algorithm tolerates the baseline fluctuations to a certain extent and adjusts the threshold accordingly.^25^ A pitfall with these approaches is that they do not thoroughly resolve the subjectivity problem during the spike recognition, and some predefined parameters are needed, *e.g.*, size of the average window, rough amplitude of the spikes or steps. Other algorithms, *i.e.*, ADEPT^26^ and second-order-differential-based calibration (DBC) with an integration method,^27^ can also predict the time points at which spikes appear. They usually require a prior rough knowledge of the spike position so that a segment containing a single spike can be singled out for further precise fitting. Furthermore, several advanced machine learning algorithms have been developed with a focus on specific tasks of processing pulse-like signals, such as denoising, feature extraction, and classification. However, machine learning, including artificial neural networks (NNs), has seldom been involved in the most essential part of signal processing in this procedure: pulse recognition.^23^ Different from the classification task, pulse recognition requires acquisition of pulse features from a noisy background in a time sequential manner, which poses a challenging task to directly adopting commonly used NN structures including convolutional neural networks (CNN) and fully connected deep neural networks. The hidden Markov model is one such attempt to detect pulses from nanopore sensing signals. However, an initial pre-processing step is required to enable the detection, and it involves the use of a user-defined criterion to determine the approximate position of current blockage events.^28^ The determination of this threshold is based on user experience and, therefore, varies from case to case.^29^ Obviously, objective algorithms are desired for isolating pulses from noisy time-sequential traces. Our recently introduced NN-based algorithm named Bi-path Network (B-Net)^29^ has addressed this subjectivity issue. B-Net is composed of two branches, each one using a residual neural network (ResNet) structure. The novelty with B-Net lies in its assignment of a different task to each branch, one counting the number of pulses in signal segments while the other measuring the features of the pulses in the segments. This design gains some inspiration from certain streams in the brain in which different pathways handle specific tasks. For instance, the ventral/dorsal streams in the visual system process the “what/where” of objects. In this way, the training process is easier for each branch and the generalization performance is increased. Unfortunately, B-Net can only predict averaged features, such as amplitude and duration, of the pulses in input data. It falls short in singling out pulses in temporal windows. A potential drawback with only obtaining average features is loss of information about the analytes.

Here, a deep learning (DL) method is proposed for pulse recognition. It is capable of predicting the start and end time points of a pulse. The duration of the pulse can, thus, be naturally obtained. The method, named pulse detection transformer (PETR), is based on a transformer architecture. Primarily, transformers have achieved state-of-the-art results in many natural language processing tasks.^30,31^ Attentional maps of transformers have been applied with outstanding success to accurately predict protein structures, an important research problem that had been open for more than 50 years.^32^ In recent years, this approach has seen increasing applications in computer vision (CV) as well.^33^ An architecture combining a transformer NN with a CNN is adopted in the present work. We focus on the application of this architecture in the field of object detection.^34^ The original application is adapted from two-dimensional (2D) object detection in bidimensional images and implemented for one-dimensional (1D) pulse detection in unidimensional temporal signals. Our method simplifies the detection pipeline by removing many hand-designed components commonly found in object detection architectures.^34^ Furthermore, we avoid complex training procedures with many independent stages in the pipeline and propose a new architecture that can be easily trained with the end-to-end philosophy that has led to the significant success in DL.^35^ Crucially, the need for subjective user-defined thresholds is eliminated.^21,22^

Specifically, we use electrical nanopore sensing signals, *i.e.*, the ionic current traces from nanopore sensors, as typical examples to evaluate PETR. Following the idioms in nanopore sensing, “spike” is usually used to refer to “pulse” as the signal. In this work, “spike” and “pulse” are used interchangeably. We will generate synthetic datasets of analyte-translocating nanopores and use them for training and validating the PETR. Afterward, we use experimental datasets of DNA- and streptavidin-translocating solid-state nanopores to further evaluate PETR by systematically comparing the results to the counterparts of B-Net and threshold-based traditional methods. Moreover, two representative algorithms known in the community are utilized to process these segments and extract features in order to demonstrate the generalized usage of the output pulse segments from PETR.

To evaluate the performance of PETR, we refer to the concept of mean average precision (mAP) from object detection in CV and adapt it to the requirements posed by nanopore translocation signals. The performance of the PETR is better for longer translocation durations. Finally, the performance for narrow translocation events can be boosted by artificially transforming them into longer translocations with larger apparent durations by means of an interpolation process.

Therefore, this work offers an objective spike recognition algorithm that delivers an accurate prediction of each spike in a temporal window from a noisy nanopore translocation trace with an mAP close to 1. PETR achieves it through returning spike segment predictions by specifying the start and end time points of each pulse in a signal window. This proposed method is expected to have a significant impact in the SMA research community since it offers a flexible generalized interface toward downstream algorithms such as feature extraction and classification.

## Results

### Model and Implementation

**Figure 1:**
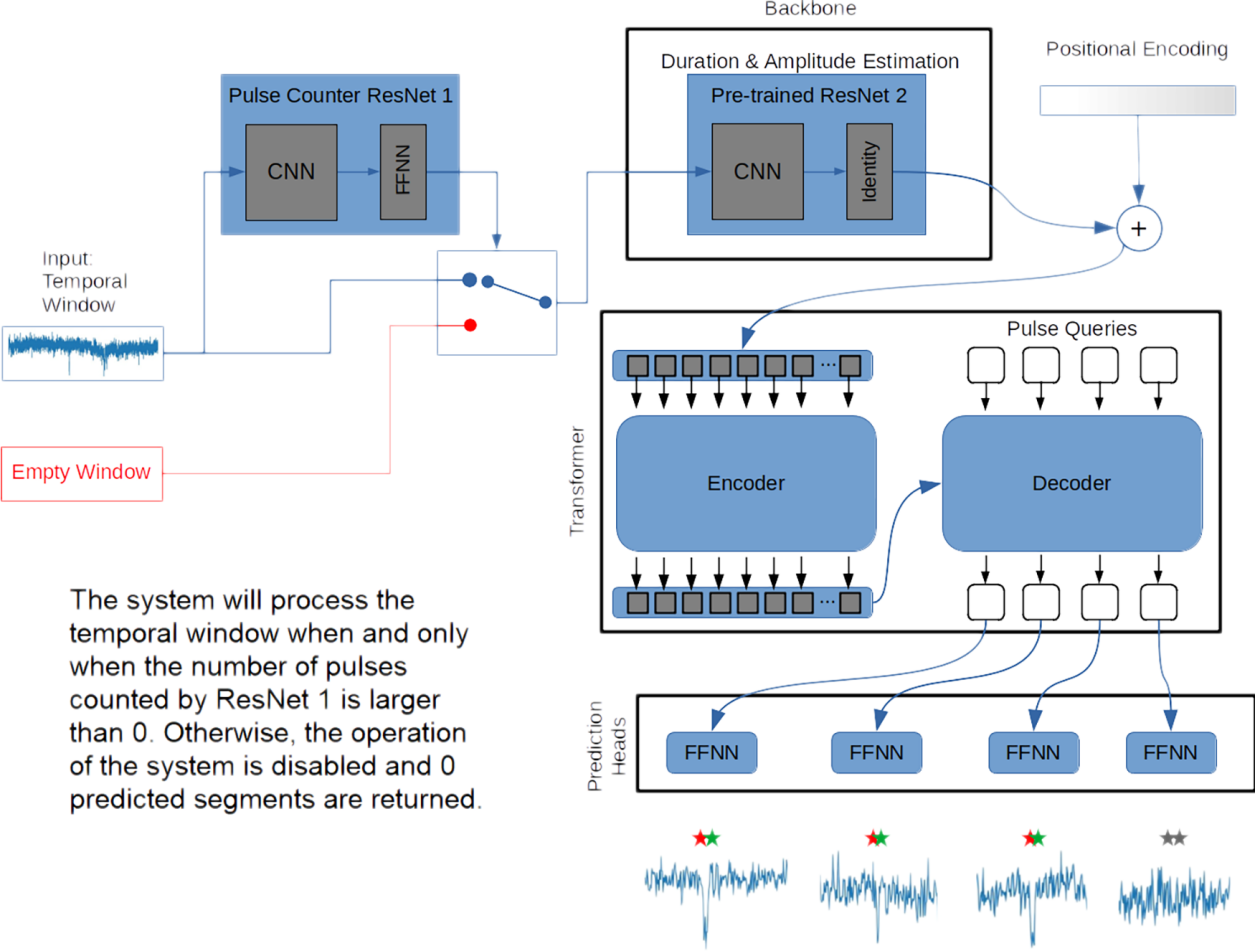
PETR as an algorithm with the capacity of detecting distinctive acute events into 1D noisy signals. It uses the feature estimation path, ResNet 2, of our previously developed B-Net model as the backbone. The backbone is a pre-trained network fine-tuned to return better 1D representations that are more adapted to the detection task. The outputs from the backbone are first added up to a 1D positional encoded vector and then passed to the transformer encoder. The transformer decoder receives a certain number of learned embeddings (called pulse queries) and returns a set of output embeddings while attending to the encoder outputs. Each decoder output is passed to an FFNN that predicts two different aspects of a detection. A detection class can be pulse (colored) or no-pulse (gray) and a bounding segment.

PETR is developed as an algorithm that can help detect characteristic events in time-sequential 1D noisy traces. This network is inspired by the model called detection transformer.^34^ The architecture of PETR is depicted in Figure 1. The network receives a 1D temporal window, which is a chunk of a noisy trace and returns a set of bounding segment predictions. The bounding segments are composed of start and end time points that predict the location of a pulse in the temporal window generated by, *e.g.*, a translocation of an analyte in a nanopore. PETR consists of four main blocks: a pulse counter, a backbone, a transformer, and a feed forward network. The original detection transformer (DETR) architecture was developed with the ultimate purpose of being readily implementable in any DL framework that provides a common CNN backbone, a clear distinction from many modern detectors.

In our case, the modularity and simplicity in the original implementation of DETR^34^ have been adopted by taking advantage of a detection model whose main property is its end-to-end trainability. We have further adapted the original architecture for 1D “images” via decoupling the original backbone and using instead the feature prediction path in our previously developed B-Net,^29^ an architecture composed of two ResNets. ResNet 1 predicts the number of pulses in a temporal window, while ResNet 2 predicts the average duration and amplitude of all the pulses in the window. Similarly, ResNet 1 in our algorithm PETR predicts the number of pulses in the input temporal window. When and only when the number of pulses is larger than zero, the complete system, *i.e.*, the backbone, transformer, and prediction heads, will process the temporal window. Otherwise, the operation of the system is disabled, and zero predicted segments are returned. ResNet 2 is used as the backbone since it has already been trained in B-Net to condense the abstract information from raw signals. The transformer encoder receives the output from the CNN in ResNet 2. It is worth noting that PETR is not an extension of B-Net. PETR’s core unit, the transformer, is totally different from B-Net. Here, only the functional components are disassembled from B-Net and reused in the PETR peripherals for convenience. One could also independently train a normal CNN as the backbone of PETR, instead of ResNet 2, and use other preprocessing techniques to filter out the blank segments without pulse instead of ResNet1. The use of a pretrained backbone in these kinds of architectures is a recurrent practice in DL.^34^

As to bounding boxes, bounding segments have been utilized in our study to reshape the concept of bounding boxes from 2D to 1D. PETR uses a detection architecture whose performance is not influenced by human conducted heuristics. The network objectively abstracts the main features from the noisy signals in order to attain the best detection experience. The self-attention mechanism of the transformer prototypes all pairwise interactions among elements in the positioned backbone output.

Finally, each output embedding from the transformer decoder is processed by feed-forward fully connected neural networks (FFNNs) that classify the pulses as present or absent and predict the start and end time points of the bounding segments. The gray bounding segments in Figure 1 are classified as absent pulses by the network, while the colored ones are classified as effectively present pulses in the window.

It is worth recalling that PETR predicts all pulses in parallel, thereby avoiding recurrence as in autoregressive models. It is trained end-to-end using a set of loss functions such as bipartite matching between predicted and ground truth pulses. When this kind of architecture predicts a bounding segment in a certain section of a trace chunk, such a prediction is not just based on the patterns nearby the prediction location but also determined by paying attention to all the surroundings inside the window. The prediction is in fact influenced by, *e.g.*, the frequency, amplitude, and duration of the pulses in all the surroundings of the prediction location.

We generate artificial datasets and use them for network training, validation, and testing. In each dataset, three important parameters are systematically varied, *i.e.*, the diameter of the analytes (15 kinds), the concentration of the analytes (20 kinds) and the duration of the translocation spikes (5 kinds). In order to measure the PETR performance, the datasets with different signal-to-noise ratios (SNR) ranging from 4 to 0.25 are also generated. In addition, two experimental datasets of λ-DNA and streptavidin-translocating solid-state nanopores are introduced to further validate the PETR fidelity. As a final demonstration of the benefits from its generalized output, two additional feature extraction algorithms are employed as use cases. Details of the data preparation can be found in the Methods section.

The training process starts by providing a random batch of temporal windows from the training data. Only temporal windows with at least one pulse are used for training. Once the total number of temporal windows is consumed in the dataset, one epoch is completed. Inside each epoch, the learning rate is set to 1 × 10^–5^, with a learning rate decay of 10 in a period of 100 epochs. We validate our model periodically by utilizing mAP, which is a widely used performance metric for object detection in 2D images.^36^ The model with the highest mAP is saved as the best representation.

An adapted mAP is used for evaluating the model. Instead of using intersection over union (IoU) as a threshold, the relative distance between the midpoint of both the predicted pulse and the ground truth is considered, referring to the duration of the ground truth pulse. A 100% threshold means that their distance is equal to the duration of the ground truth pulse. The associated mAP is calculated as an average by varying the relative distance thresholds from 100 to 400%, with steps of 10%. Any matched pair of the predicted pulse and the corresponding ground truth with smaller distance than such threshold is considered a true positive. Compared to the IoU thresholds adopted in the standard mAP, the adopted thresholds are more tolerant. However, the adapted mAP in the pulse detection scenario appropriately reflects the necessary requirements in order to catch nanopore translocation events in trace windows. Furthermore, coverage, which is defined as the total number of true positives divided by the total number of ground truth pulses, is calculated as another metric for pulse detection performance. In addition, the duration error as well as the start and end time errors are computed as indirect performance measures of PETR.

### Performance on the SNR = 4 Dataset

**Figure 2:**
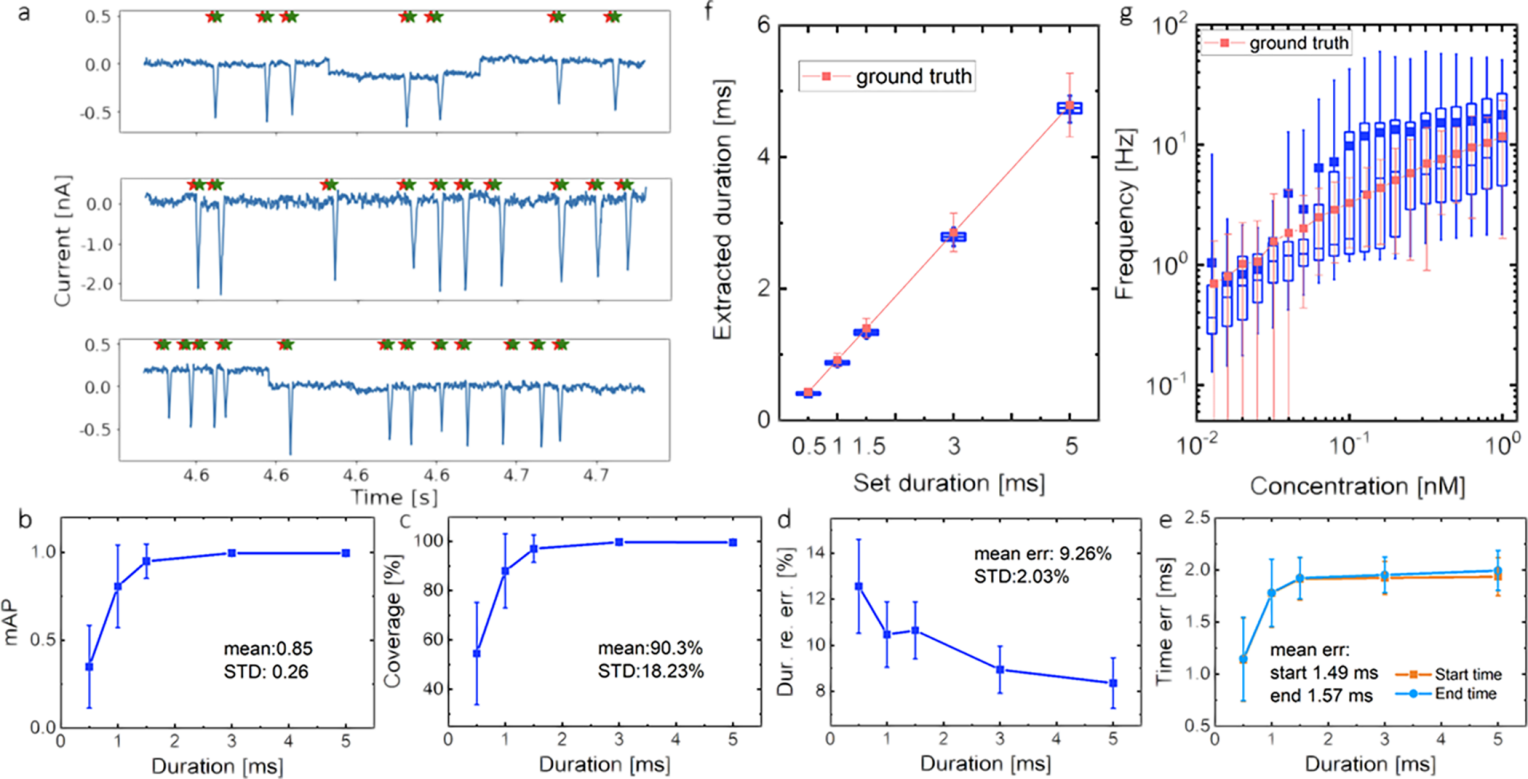
Results of PETR processing the SNR = 4 dataset. (a) Typical examples of the PETR output. In the current trace segment, PETR predicts the start and end time points of the spikes, marked as red (start) and green (end) stars, respectively. (b) mAP and (c) coverage of the spike detection at different durations. (d) Relative errors of predicted duration and (e) errors of start and end time points for spikes at different durations. In each sub-figure, the corresponding overall mean and standard deviation on the entire dataset are included. (f) Box chart showing the distribution of extracted durations for the spikes at different set durations in the dataset. (g) Box chart showing the distribution of spike appearance frequency for the signal generated at different concentrations of analytes. In (f) and (g), pink dots (average values) with error bars (spread) mark the corresponding quantities of the ground truth, indicating an excellent agreement between PETR predictions and ground truths.

The PETR performance is evaluated for our artificially generated dataset with SNR = 4. Several typical examples of the detected spikes are depicted in Figure 2a with the start and end time points respectively marked in red and green stars predicted by our model. The adapted mAP of the detection of the spikes with different widths (*i.e.*, translocation duration) is displayed in Figure 2b. PETR returns an almost-perfect detection with mAP ≈ 1 for the long spikes, *e.g.*, duration ≥ 1.5 ms. However, the detection precision for spikes shorter than 1.5 ms decays rapidly with decreasing duration. The overall mAP on the entire dataset is 0.85. Evaluating our model by employing the standard mAP produces results with an mAP of ≥0.3 for spikes of 5 ms duration, which is comparable to detectors used in other application scenarios.^34,37−39^ Details of the performance measured by the standard mAP can be found in Supporting Information (SI).

The coverage shows a similar trend with the spike duration in Figure 2c. PETR has rarely missed spikes with duration longer than 1.5 ms, as reflected by a coverage close to 1 in the figure. The coverage drops for shorter spikes, though it can still reach 60% for durations equal to 0.5 ms. Thus, the overall coverage of PETR is above 90% on the entire dataset.

The relative errors of the predicted duration by the start and end time points from PETR are shown in Figure 2d for different duration spikes. The relative error is smaller for longer spikes and the average error on the entire dataset is below 9.3%. Furthermore, the absolute errors on the predicted start and end time points are displayed in Figure 2e, showing an average value of ∼1.5 ms.

For nanopore translocation, three features are most widely discussed and studied for a spike, *i.e.*, duration, appearance frequency, and amplitude.^40,41^ The first two can be naturally obtained by PETR as the by-products of the detection process. The box charts of the predicted duration and frequency of the spikes summarized in Figure 2f,g represent their statistical distributions on corresponding setting parameters in the data generation, *i.e.*, set duration and analyte concentration, respectively. For a better comparison, the average values of the ground truth are also shown as dotted lines with error bars in the same figures. It is found that the predicted values coincide well with the respective ground truths, indicating that PETR can efficiently detect the spikes with high accuracy.

An important reason for the good recognition performance is that PETR naturally resists the baseline fluctuations. Two kinds of fluctuations are commonly seen in single-molecule detection signals and are involved in the artificially generated datasets as well: slow drift and sudden jumps. The recognition of pulses by PETR is based on the pulse features and their differences from the background noise, which is insensitive to the baseline level shift and, thus, resistant to the slow drift of baseline. Sudden jumps generate rapid changes of a signal, which are similar to the rising or falling edges of pulses. One of the main features of a pulse, the target of PETR, is that its rising and falling edges appear in pairs, which rarely happens with sudden jumps. Even in the atypical scenario of a rising edge and a falling edge occurring together with a similar timing to the one found in a normal pulse in a window, the network will not detect such a fluctuation as a pulse. PETR acquires not only the timing of pulses but also the statistical distribution behind their main morphological features. This feature makes PETR immune to sporadic atypical fluctuations in the signal. Several examples showing how PETR is immune to baseline fluctuations can be found in the SI (Figure S3).

### Datasets with Different SNRs

**Figure 3:**
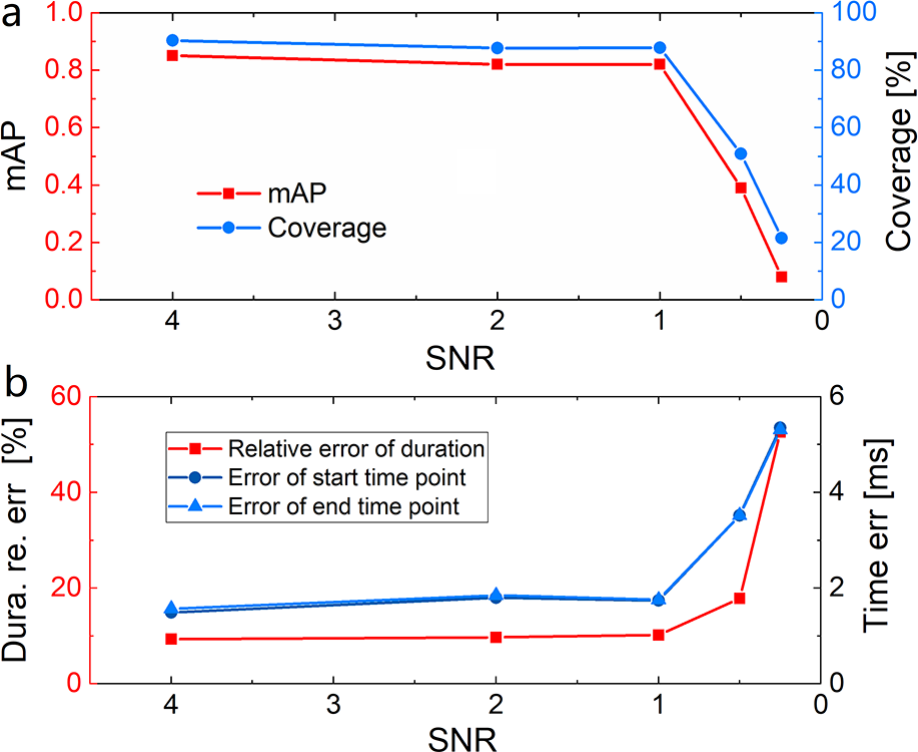
PETR performance for the datasets with different SNRs. (a) mAP and coverage of the spike detection at different SNRs. (b) Relative error of the predicted duration and error of the start and end time points at different SNRs.

The PETR performance is also evaluated on our artificially generated datasets with SNRs other than 4. The overall mAP and coverage on all the datasets with SNR ranging from 4 to 0.25 are summarized in Figure 3a. Furthermore, the relative error of predicted duration and the absolute error of the start and end time points for the different SNR datasets are displayed in Figure 3b. Details about these parameters, distributed on different setting variables in the data generation process, such as duration, analyte concentration, and analyte size, for each dataset can be found in the SI.

It can be clearly inferred from the charts that the PETR performance does not decay substantially even when SNR decreases from 4 to 1. The mAP for SNR ≥ 1 stays above 0.8, and the coverage stays above 87%. The relative error of duration is lower than 10.2%, and the error of predicted start and end time points is smaller than 1.8 ms. The detection and prediction abilities of PETR sharply fall for SNR < 1. However, even for the worst case with SNR = 0.25, PETR can still capture 20% of the spikes and the relative error of duration is only ∼50%. If an mAP of >0.8 and coverage of >85% are set as the criteria for an accurate processing at 10 kHz sampling rate, the shortest pulses that can be detected accurately are 1, 1, 1, and 5 ms for SNR = 4, 2, 1, and 0.5, respectively. These results indicate that PETR has an outstanding generalization ability to adapt to a wide range of noise space.

In this work, SNR-specific networks are trained individually for the five different SNRs studied instead of training one common network for all data with various noise levels. This design is adopted by considering the properties of PETR as a machine learning model: the recognition mechanism is based on the acquisition of the statistical distribution behind the pulse properties of the signal and the surrounding background noises as the context. The differences between the pulse and noise are implied in the signal–noise context. Each SNR carries a distinct signal–noise relationship, which naturally requires a specific set of trained weights to achieve the best performance. On the contrary, training a common network for all SNR can gain an added degree of automatization for data processing but largely at the expense of detection performance. Obviously, this approach does not bring any benefit. Furthermore, the background noise is usually a stationary stochastic process from an experimental perspective. Thus, the statistical properties of noise do not change considerably in one record of the current trace or even during the entire measurement period as long as the experimental conditions, *e.g.*, bias voltage, analyte concentration, temperature, and surface cleanness, are stable. In practice, well-trained networks for different SNRs can cover most of the application scenarios. In order to compensate for the loss of the degree of automatization due to the use of SNR-specific networks, an extra pre-processing section can be added to detect the rough level of SNR. Alternatively, it is also possible to label a fraction of the target dataset and use it for determining the best-performing model. While viable, this last option is expensive and tedious.

### Compensation of Short Duration Spikes

The results shown in Figures 2 and 3 point to the critical dependence of PETR detection precision and coverage on the spike width (duration). If the duration of a spike is too short, the number of sampling points can be too limited to ascertain its recognition from the noisy background. For example, using a sampling rate of 10 kHz generates five sampling points for a 0.5 ms spike and 50 sampling points for a 5 ms spike. In other words, it is the number of sampling points, instead of the absolute duration time span, that determines the detection performance of PETR. Therefore, increasing the sampling rate of the data acquisition, as well as the bandwidth of the experimental setup, can enhance the detection performance for the short spikes. From a signal-processing perspective, interpolation can reach a similar performance improvement. This approach is validated using a generated dataset (SNR = 4) with 0.5 ms spikes by linearly interpolating with nine points in between each two original adjacent points. Hence, the apparent effective spike duration of PETR becomes 10 times longer, *i.e.*, 5 ms. However, the interpolation can alter the noise characteristics and thereby interfere with the decision-making of PETR. In order to compensate for this adverse effect, a small amount of artificially generated noise, which has the same components in the power spectrum as the ones in the training dataset, is added to the interpolated data. By adding a noise component of half of the original amplitude in the signal (measured on a small segment of input signal traces), which only increases the total noise power by 1^2^ + 0.5^2^ = 1.25 times, the resultant SNR is only slightly worsened from 4 to 4/ = 3.58. However, this small amount of added noise can significantly boost the detection performance of PETR because the signal–noise characteristics become similar to that of the original training case with SNR = 4.

There are prerequisites for the interpolation and compensation. First, the bandwidth of the system is large enough for the target signal, *i.e*., the pulses, and the sampling frequency to discretize the signal is sufficiently high in accordance to the Nyquist theorem. Second, the general knowledge of the noise characteristics in the system should be acquired, such as the noise components that dominate in different frequency ranges. Here, the noise characteristics of nanopores have been studied and noise models are well established.^42,43^ Hence, the original signal with its raw data already contains sufficient information for correct recognition. However, the data points are often too few for PETR to perform a sensible detection of a pulse. In detail, the number of data points in the pulse is simply insufficient for the convolutional kernel to process them or for the transformer to pay enough attention to them. Consequently, PETR does not perform well on these short-duration-pulse traces. Therefore, interpolating points that contain a small amount of noise with similar characteristics to the noise present in the original signal can alleviate the challenge with limited data points and improve the detection performance. It is important to clarify, however, that the interpolating samples do not generate additional information.

**Figure 4:**
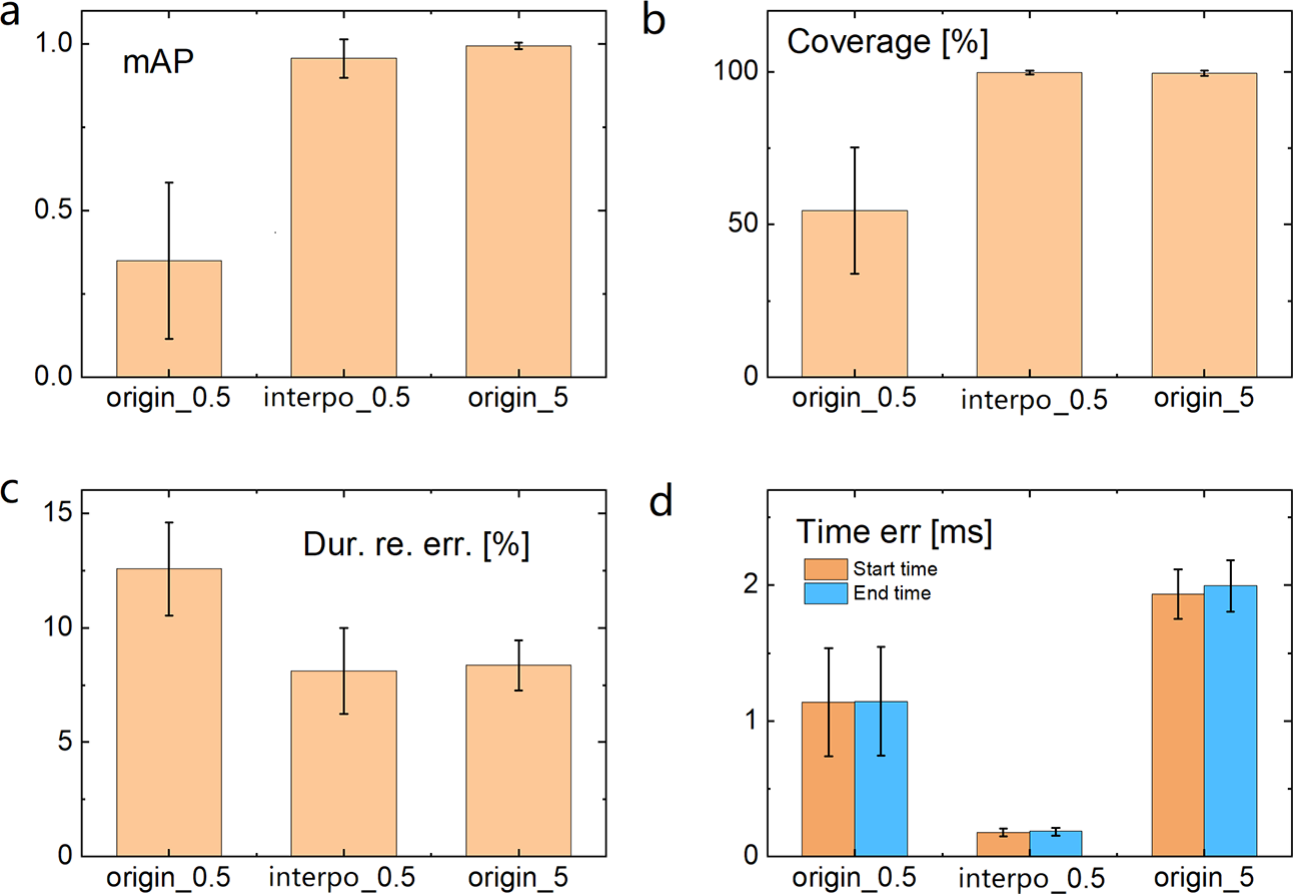
Comparison of the PETR performance with interpolation. (a) mAP, (b) coverage, (c) relative error of duration, and (d) errors of the start and end time points, for the interpolated 0.5 ms duration data in comparison with those for the original 0.5 and 5 ms data.

Both mAP and coverage of the PETR spike detection for the original and interpolated 0.5 ms data are compared in Figure 4. The detection performance for the original 5 ms data is also shown as a reference. It is clear that both mAP (Figure 4a) and coverage (Figure 4b) are significantly improved after data interpolation, and they are comparable to those for the original 5 ms data. It indicates that after the interpolation with a 10-fold increase in number of points, PETR “sees” the data as if they came from the 5 ms spikes. The relative error of duration for the interpolated data is also similar to that of the 5 ms data (Figure 4c). Since the errors of the start and end time points depend on the number of points in a spike instead of the absolute value of duration time, the higher the sampling rate equivalently achieved by interpolation, the lower is the absolute error in time (Figure 4d). Details of these parameters distributed on different setting variables can be found in the SI.

### Performance Evaluation on Experimental Datasets

**Figure 5:**
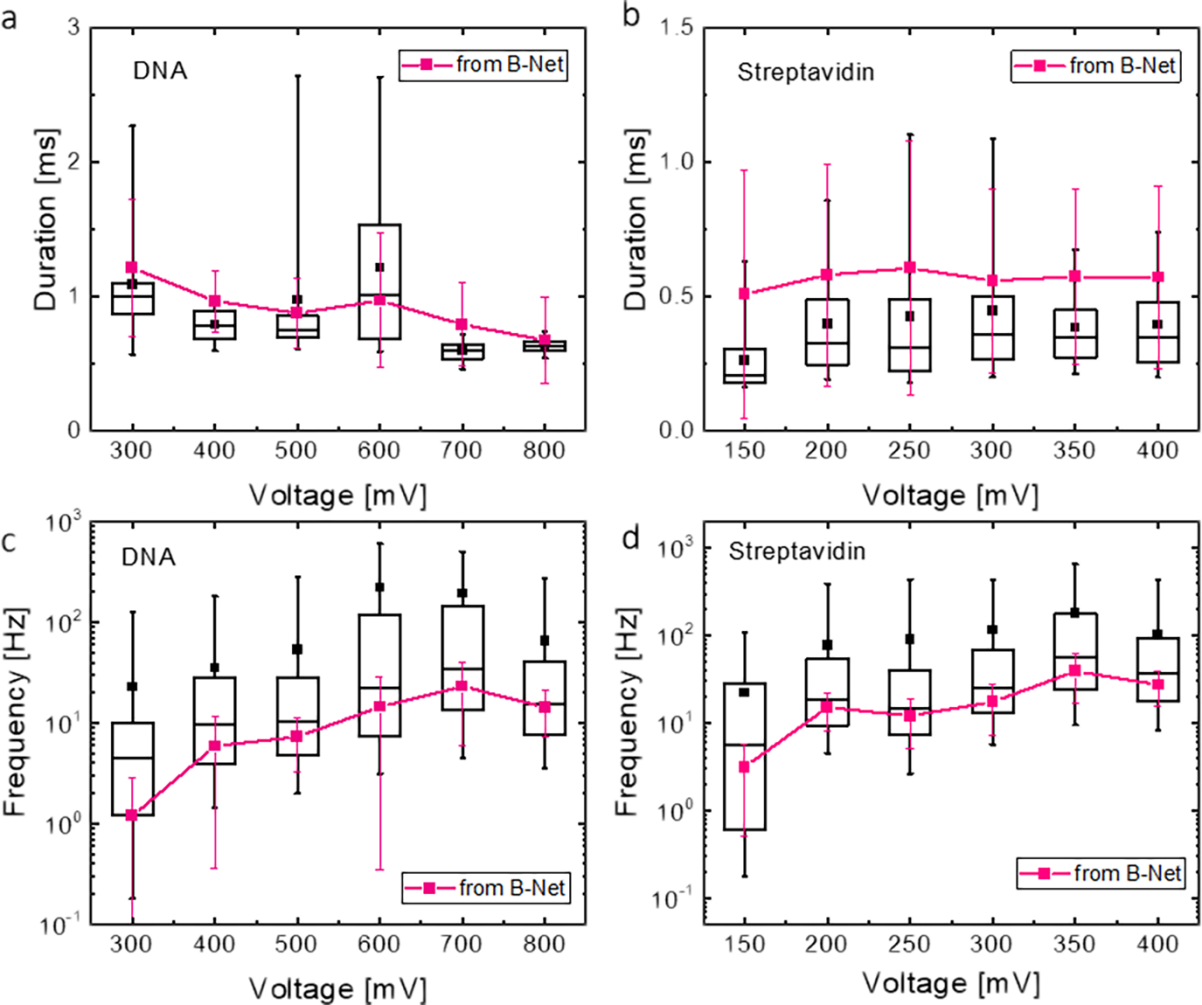
Results of PETR processing the experimental datasets. Box charts showing the PETR distribution of extracted duration at different bias voltages for (a) DNA and (b) streptavidin translocation data. Box charts showing the distribution of spike appearance frequency at different bias voltages for (c) DNA and (d) streptavidin translocation. The pink dot-on-lines (average values) with error bars (spread) in each figure display the corresponding results from our previously developed B-Net algorithm.

PETR is also applied to processing experimental data from the translocation of λ-DNA and streptavidin in solid-state nanopores. The duration and appearance frequency of translocation events at various bias voltages are shown in Figure 5. The latter shows an upward trend with increasing voltage in accordance with the physics of the capture process. Higher voltage offers larger capture area, thereby yielding higher frequency.^41^ The observed constant duration of streptavidin translocation at the bias voltages used is attributed to the limited bandwidth of signal acquisition in our experiment; it is readily conceivable that a high translocating speed of small molecules such as protein can lead to a sharp and featureless spike.^44,45^ The duration does not display a monotonous trend in the DNA translocation data, which may result from complicated interactions between long and densely-charged DNA and nanopore.^44,46^ The traditional method in which different multiples of noise level are utilized as thresholds for spike detection yields diversified results on both frequency and duration (SI, Figure S15). In sharp contrast, physics-plausible, stable, and consistent trends are obtained by PETR based on the same experimental data (SI).

The PETR results agree satisfactorily well with those based on our previous algorithm B-Net^29^ (Figure 5). Both algorithms give consistent results even for the variation details of these two parameters, duration and frequency of the translocation events, along the different bias voltages. It is important to emphasize that the two algorithms have completely different objectives along with distinct NN structures and output formats. The unanimous results strongly support that PETR is effective and reliable. Nonetheless, systematic deviations between PETR and B-Net appear. PETR generally predicts a shorter duration (Figure 5a,b), but a higher appearance frequency (Figure 5c,d) than what B-Net gives. These observations consistently point to the ability of PETR to capture more spikes of relatively smaller amplitudes and shorter durations than B-Net. That PETR raises the average appearance frequency and lowers the average duration can be related to the essential difference in the core tasks of the two architectures, PETR versus B-Net. As mentioned, B-Net predicts averaged properties of the pulses in each input segment. This network does not detect individual pulses, and it has a focus on the more obvious spikes with larger amplitudes and longer durations. In contrast, PETR detects pulses according to their distinct features from the noisy background, *i.e.*, the context around the pulses, and treats pulses as individual entities. By this mechanism, PETR could catch smaller spikes.

### Demonstration of Generalized Output

The primary objective of the PETR algorithm is to single out all spikes in a time-sequential trace. Being endowed with the largest flexibility, the PETR outputs in the form of spike segments can be later adopted and processed by other algorithms for different purposes, including extracting the features and then classifying and correlating them to the physicochemical properties of the analytes. To demonstrate the general utility of PETR, two established algorithms, ADEPT^26^ and DBC,^27^ are adopted to post-process the spike segments singled out by PETR. In short, current segments containing single spikes objectively recognized from PETR are directly used as raw materials for ADEPT and DBC to more precisely determine the spike amplitude.

The ADEPT algorithm is based on the pulse response of the nanopore system according to its equivalent circuit. Rising and dropping periods in spikes are fitted by several exponential functions with different time constants, leading to the extraction of duration and amplitude of the spikes. In the DBC method, the spikes are first fitted by a Fourier series for smoothing. The second-order derivative of the smoothed waveform is then calculated for determination of its extrema. The positions of the two largest minima are then correlated to the start and end time points of the translocation events. Finally, the amplitude is extracted by considering the area enclosed by the spikes referring to the baseline. Detailed processing flows for both algorithms can be found in the SI with typical examples.

The extracted amplitude and duration of the spike segments by means of the ADEPT and DBC methods show physics-plausible and stable results (see SI, Figure S17). The spike segments result from the PETR detection data of the λ-DNA and streptavidin translocation datasets. In detail, the spike amplitude increases with increasing bias voltage, which is reasonable since higher voltage induces a larger ionic current through the nanopore. The spike duration of λ-DNA and streptavidin follows similar trends as those extracted directly by means of PETR for different bias voltages. The comparisons confirm that the results from both methods, as well as the spike segments singled out using PETR, are reliable.

## Discussion

The detection of pulses using PETR is based on the acquisition of the signal–noise characteristics of the input by the NN. With a synergic consideration of the abrupt changes of corresponding signal properties during the pulses and the background noise surroundings as the context, PETR can distinguish the differences between the pulses and the noise and recognize them. This approach brings a completely different strategy from the traditional threshold-based methods, since the latter only consider the highly simplified amplitude information. Therefore, the PETR results are consistent and free from user-defined parameters. Furthermore, PETR can automatically adapt to complicated real-world scenarios with baseline fluctuations, noise level alternations, pulse amplitude variations, *etc.*, thereby yielding stable and reliable outputs. Thus, PETR, unlike the traditional methods, is characterized by the ability of generalization. The strategy of PETR in dealing with complex problems with pulsed signals also determines that its generalization ability highly depends on the characteristics of the pulses and the background noise as well as their differences. Thus, PETR will return a better performance for datasets with more similar noise–signal characteristics to the training dataset. Moreover, the detection performance of PETR for the interpolated data can be enhanced by adding a small amount of noise with the same spectrum as the one found in the training dataset.

Compared with our previously developed B-Net, PETR has an entirely different aim. Instead of extracting the features of pulses, it focuses on isolating individual pulses from a noisy background in a time-sequential trace. From the perspective of the network structure, a well-adopted practice in object detection is to use the pre-trained features of a CNN as a pre-processing (backbone) platform.^34^ From its convolutional architecture, this pre-processing platform provides the system with the needed inductive bias to capture important features existent in signals found in nature, such as pulse-like signals from molecular translocating events.

PETR utilizes the CNN part of the ResNet 2 in the B-Net as its backbone. This section of the network contributes important features about the signals to the system, playing a pre-processing role of information abstraction and condensation. However, the essential process of acute pulse recognition and localization is realized by the transformer structure. The transformer plays the role of a memory bank, which is not affected by inductive bias as the backbone. The transformer focuses on learning the statistical distribution in the context of the time-sequential features delivered by the backbone. The transformer uses the backbone features to memorize the semantic structures of the different situations present in the signals. For instance, if a pulse of certain duration is found in a location, it is highly likely that some pulses with a similar duration could be found in the vicinity of such a detection, since the signals used for training have such properties.^47^ If the network has detected some repeated patterns of pulses in a region of the temporal window, this observation will help the network to take a decision about similar trends in other regions of such a window. All these semantic features about the structure of the signals are memorized by the transformer by referring to the pre-processing features provided by the backbone. It is worth noting that the achieved performance of PETR relies on the fact that the backbone efficiently extracts and condenses basic features in the signals and that the transformer acquires the semantic properties of pulses, such as positions and appearance frequency, by considering the context in the time-sequential signal.

## Conclusions

With the transformer structure as its base, PETR can successfully single out pulses from a noisy background in nanopore-sensing signals. The typical machine learning end-to-end training strategy of PETR avoids user-defined thresholds and, thus, the subjectivity of users. PETR is first trained on generated datasets with different SNR levels. It is further validated by both generated and experimental datasets. Outstanding PETR detection performance is demonstrated to be achievable even for low SNR data. As the detection performance is largely influenced by the number of sampling points in each pulse, a simple linear interpolation can significantly improve the detection precision and coverage for short pulses. PETR is proven to be a generalized method for spike detection and offers a powerful tool for processing signals from various single-molecule events in SMA. It further acts as an important link in the pipeline of pulse-like signal processing by offering a seamless connection to the downstream algorithms.

## Methods

### Pulse Detection Transformer

As is the case for the original DETR model by Carion *et al.*(34) PETR possesses a reasoning-like behavior that predicts each bounding segment by taking into account a global context in the entire temporal window. At the end, the system produces all predictions in parallel ,and each bounding segment is predicted based on the global context surrounding such a prediction.

### Pulse Counter

The pulse counter path (ResNet 1) in our previously developed B-Net was used to count the number of pulses in the temporal window to be processed by the system. The B-Net architecture uses two ResNets that are pre-trained for regression tasks—ResNet 1 is trained for pulse counting in a trace chunk, and ResNet 2 is trained for averaged duration and amplitude prediction of the pulses inside such a chunk. As shown in Figure 1, the system would process the temporal window only if the number of pulses counted by ResNet 1 was more than zero. Otherwise, the window was discarded, the operation of the system disabled, and zero predicted segments returned. This section of the network was only involved in testing activities. ResNet 1 did not take part in either network training or validation.

### Backbone

The feature prediction path (ResNet 2) of our previously developed B-Net was used as the backbone in PETR.^29^ The original pre-trained B-Net uses the ResNet18 architecture. The FFNN layers in ResNet 2 were replaced by identity layers that only passed the input to the output without modification. Hence, the output returned by the backbone was the flattened version of the output of the convolutional section in ResNet 2.

In its original function, the B-Net was trained to predict characteristic features inside a temporal window extracted from a noisy trace. Based on the DL architecture of the B-Net, with its end-to-end training philosophy, it is highly feasible that the convolutional section in each ResNet acquired important features in the statistical structure of the translocating pulses in the signal. These features turned out to be highly effective for training the entire detection system we are introducing here. ResNet 2 was fine-tuned for the detection task, and the learning rate applied to it was a constant but a smaller value than the one used for the rest of the network.

### Transformer

As its name alludes, transformer architectures transform one sequence at its input into another sequence returned by its output. Even though transformers materialize the state of the art in today’s machine learning sequence processing, these architectures completely dispense recurrence, thanks to their attention mechanisms that process sequences integrally in parallel.^48^

The output from the backbone is a sequence of vectors. Each vector in the sequence has a number of channels (num_channels). The sequence is processed by a kernel_size = 1 1D convolution that reduces the number of channels in each vector in the sequence to hidden_dim. In our case, since we used ResNet 18 in the backbone, num_channels = hidden_dim = 512, there was no dimensionality reduction.

Afterward, the transformer encoder added the 1D positional embedding vector to the input sequence, and the resulting positioned sequence was passed through *Nenc* successive encoder layers composed by a multi-head self-attention (MHSA) layer followed by an FFNN layer as residual stages were bypassed by skipping connections.

The transformer decoder received a sequence of pulse queries, which was a set of learned embedding vectors. Each embedding vector had hidden_dim components. First, the embedding vectors were passed to an MHSA, then the output from this one was passed to a multi-head attention (MHA) layer. The MHA layer also paid attention to the outputs from the transformer encoder. The output from this MHA layer was passed to a final FFNN. The transformer decoder had Mdec layers repeating this processing pipeline.

### Feed-Forward Fully Connected NN

Finally, the outputs from the last layer of the transformer decoder were passed to two FFNN modules. One of these modules classified the pulse queries as present or absent pulses in the trace chunk, while the other predicted the bounding segments for each classification, *i.e.*, predicted the location of the pulse in the temporal chunk extracted from the noisy trace.

### Pulse Detection and Prediction Losses

In each single pass through the decoder, PETR predicted a fixed-size set of *N* pulses in a temporal window in a noisy trace. *N* was a chosen parameter, and it was larger than or equal to the maximum number of translocation events produced inside a temporal chunk in the complete dataset. The system found the best bipartite matching between predictions and ground truths based on class, position, and size. The matching cost took into account the class prediction, which was an existent or non-existent pulse, and the similarity between the predicted and the ground truth bounding segments.

Once the matching was done, each prediction was assigned to a ground truth bounding segment and the system now could compute the Hungarian loss for all the pairs matched in the previous step. The Hungarian loss is a combination of class prediction and bounding segment losses, *i.e.*, this combines a class cost, a bounding segment cost, and an intersection over union cost.

## Data Preparation

### Data Generation

The artificially generated data was composed of three parts: (1) randomly appeared translocation spikes, (2) background noise, and (3) baseline variations. The baseline current level, random properties of a translocation, current blockage amplitude, and the background noise spectrum were all determined using our established physical models with given corresponding parameters such as geometry of nanopore and analytes, electrolyte concentration, analyte concentration, and bias voltage. In signal generation, both the diameter and thickness of the nanopores were fixed to 20 nm. Typical experimental conditions were selected, including a bias voltage of 300 mV, a 100 mM KCl electrolyte, and −0.02 C/m^2^ surface charge density. Three parameters in the signal generation program, *i.e.*, the diameter of translocating analytes, the concentration of the analytes, and the duration of translocation, were systematically varied in each dataset. In each dataset, the diameter of the nanospheres varied from 3 to 17 nm with a 1 nm step (15 different values). The concentration of the nanospheres varied from 0.01 to 1 nM, changing in logarithmic scale (20 different values). The duration of the translocation was directly assigned to 0.5, 1, 1.5, 3, and 5 ms (five different values). In addition, the SNR was varied from 0.25 to 4 (five different values). Each dataset was composed of 1500 traces with combinations of different values of these three varying parameters. It is worth noting that the SNR is defined as the ratio of spike amplitude to the peak-to-peak value of the background noise. Details of the data generation are available in the literature.^29^ We provide training, validation, and testing datasets available online for SNR = 4 as well as testing datasets available for all the SNRs.^49^

### Experimental Data

λ-DNA and streptavidin were selected as two typical examples of the translocating analytes, representing the long strand-shaped and sphere-shaped objects, respectively. Electrical measurements were controlled using a patch clamp amplifier (Axopatch 200B, Molecular Device Inc.). The ionic current was converted to digital signal by Axon Digidata 1550A (Molecular Device LLC.) and recorded by software Axon pCLAMP 10 (Molecular Device LLC.). The translocation signal was measured under six different bias voltages for both λ-DNA and streptavidin. The λ-DNA translocation was measured in a 10 kHz sample rate with 2 kHz analog bandwidth, while the streptavidin translocation was detected at 20 kHz sampling frequency with a 10 kHz bandwidth. All the datasets have been published in Zenodo.^49^

## Training and Validation

Training was conducted using artificially generated traces as described above. For this work, five datasets, each with a different SNR, were used to train five different instances of the same detector. In the training process, we split each 20 s trace in temporal windows of 0.5 s. Accordingly, we ended up with 60,000 windows per dataset. Datasets for testing had traces of 10 s, *i.e.*, we ended up with 30,000 temporal windows per dataset for testing purposes.

The training process consisted of providing a random batch of temporal windows from the training data. Only temporal windows with at least one pulse were used for training. Empty windows were discarded for training purposes. Therefore, ResNet 1 was not used during training. Once the total number of temporal windows had been consumed in the dataset, one epoch was completed. Inside one epoch, a learning rate of 1 × 10^–5^ was adopted, with a learning rate decay of 10 in a period of 100 epochs. The number of epochs used to train a model instance depended on the level of noise in the training dataset. The batch size was of six temporal windows in all the cases.

Validation was conducted periodically, first after epoch number 50, then after epoch number 100, and from then every five epochs, *i.e.*, after epochs number 105, 110, 115, and so on. We validated our model by utilizing standard mAP, which is a widely used performance metric for object detection in 2D images.^36^ The model with the highest mAP was saved as the best representation.

For evaluating (testing), the adapted mAP was used. In our case, instead of using IoU as a threshold, we used the relative distance between the midpoint of both, predicted the ground truth segments, and referred to the length of the ground truth segment. We computed the mAP by considering relative distance thresholds between 100% and 400% with steps of 10%. A threshold of 100% means that the distance between the two segments, predicted and ground truth, is equal to the length of the ground truth segment. Likewise, a threshold of 400% means that such distance is 4 times the ground truth segment length. Any pair of matched pulses, predicted and ground truth, with shorter distance than such thresholds was considered as a true positive. Even when such thresholds could seem too tolerant compared to the IoU thresholds adopted by the detection community,^1^ in the nanopore translocation application scenarios, our adapted mAP adoption for testing appropriately reflected the requirements at time of catching nanopore translocation events in trace windows. Coverage is another important performance metric, which is defined as the total number of true positives divided by the total number of ground truth segments. We also computed the duration error and the start and end time errors. The duration error is the relative difference between the predicted and ground truth segment lengths relative to the ground truth segment length. The start/end time error is the difference, in milliseconds, between the predicted and the ground truth of start/end time. Finally, we computed the average and standard deviation of all these metrics for each duration in the test datasets.

It is important to highlight that during testing, for the computation of the adapted mAP, empty windows were discarded since mAP was inconsistent for scenes without objects. ResNet 1 was used to discard empty windows when the model was confronted with experimental data.

## Supporting Information Available

Mean average precision and our adaptation; performance of the PETR on dataset with various SNRs; performance of the PETR on the interpolated dataset; performance of extraction by using standard mAP; comparison with B-Net and traditional method; details about DBC and ADEPT (PDF)

## Notes

All data are publicly available at https://zenodo.org/record/6901525. Source data are provided with this paper. All codes are available at https://zenodo.org/record/6902557.

## Footnotes

1 For validation, we use the original mAP metric used in CV. AP at IoU = 0.50:0.05:0.95 means mAP computed using IoU between 0.5 and 0.95 at steps of 0.05.^21^

## References

[ref1] Akkilic N.; Geschwindner S.; Höök F. Single-Molecule Biosensors: Recent Advances and Applications. Biosens. Bioelectron. 2020, 151, 111944 10.1016/j.bios.2019.111944. 31999573

[ref2] Liu S.-C.; Ying Y.-L.; Li W.-H.; Wan Y.-J.; Long Y.-T. Snapshotting the Transient Conformations and Tracing the Multiple Pathways of Single Peptide Folding Using a Solid-State Nanopore. Chem. Sci. 2021, 12, 3282 10.1039/D0SC06106A. 34164097 PMC8179386

[ref3] Liu K.; Chen X.; Kaiser C. M. Energetic Dependencies Dictate Folding Mechanism in a Complex Protein. Proc. Natl. Acad. Sci. 2019, 116, 25641–25648. 10.1073/pnas.1914366116. 31776255 PMC6925980

[ref4] Schmid S.; Stömmer P.; Dietz H.; Dekker C. Nanopore Electro-Osmotic Trap for the Label-Free Study of Single Proteins and Their Conformations. Nat. Nanotechnol. 2021, 16, 1244–1250. 10.1038/s41565-021-00958-5. 34462599

[ref5] Tripathi P.; Firouzbakht A.; Gruebele M.; Wanunu M. Direct Observation of Single-Protein Transition State Passage by Nanopore Ionic Current Jumps. J. Phys. Chem. Lett. 2022, 13, 5918–5924. 10.1021/acs.jpclett.2c01009. 35731125

[ref6] Lu S.-M.; Li M.-Y.; Long Y.-T. Dynamic Chemistry Interactions: Controlled Single-Entity Electrochemistry. J. Phys. Chem. Lett. 2022, 13, 4653–4659. 10.1021/acs.jpclett.2c00960. 35604854

[ref7] Unwin P. Concluding Remarks: Next Generation Nanoelectrochemistry – Next Generation Nanoelectrochemists. Faraday Discuss. 2022, 233, 374 10.1039/D2FD00020B. 35229863

[ref8] Howorka S.; Movileanu L.; Braha O.; Bayley H. Kinetics of Duplex Formation for Individual DNA Strands Within a Single Protein Nanopore. Proc. Natl. Acad. Sci. 2001, 98, 12996–13001. 10.1073/pnas.231434698. 11606775 PMC60813

[ref9] Ying Y.-L.; Cao C.; Long Y.-T. Single Molecule Analysis by Biological Nanopore Sensors. Analyst 2014, 139, 3826–3835. 10.1039/C4AN00706A. 24991734

[ref10] Schreiber M.; Wei A.; Yuan A.; Gaut J.; Saito M.; Salkoff L. Slo3, a Novel *p*H-Sensitive K^+^ Channel from Mammalian Spermatocytes. J. Bio. Chem. 1998, 273, 3509–3516. 10.1074/jbc.273.6.3509. 9452476

[ref11] Ivankin A.; Henley R. Y.; Larkin J.; Carson S.; Toscano M. L.; Wanunu M. Label-Free Optical Detection of Biomolecular Translocation through Nanopore Arrays. ACS Nano 2014, 8, 10774–10781. 10.1021/nn504551d. 25232895 PMC4212781

[ref12] Chen M.; Lu S.-M.; Peng Y.-Y.; Ding Z.; Long Y.-T. Tracking the Electrocatalytic Activity of a Single Palladium Nanoparticle for the Hydrogen Evolution Reaction. Chem. – Eur. J. 2021, 27, 11799–11803. 10.1002/chem.202101263. 34101910

[ref13] Ying Y.-L.; Long Y.-T. Nanopore-Based Single-Biomolecule Interfaces: From Information to Knowledge. J. Am. Chem. Soc. 2019, 141, 15720–15729. 10.1021/jacs.8b11970. 31509414

[ref14] Derrington I. M.; Butler T. Z.; Collins M. D.; Manrao E.; Pavlenok M.; Niederweis M.; Gundlach J. H. Nanopore DNA Sequencing with Mspa. Proc. Natl. Acad. Sci. 2010, 107, 16060–16065. 10.1073/pnas.1001831107. 20798343 PMC2941267

[ref15] Brinkerhoff H.; Kang A. S.; Liu J.; Aksimentiev A.; Dekker C. Multiple Rereads of Single Proteins at Single–Amino Acid Resolution using Nanopores. Science 2021, 374, 1509–1513. 10.1126/science.abl4381. 34735217 PMC8811723

[ref16] Yusko E. C.; Bruhn B. R.; Eggenberger O. M.; Houghtaling J.; Rollings R. C.; Walsh N. C.; Nandivada S.; Pindrus M.; Hall A. R.; Sept D.; Li J.; Kalonia D. S.; Mayer M. Real-Time Shape Approximation and Fingerprinting of Single Proteins Using a Nanopore. Nat. Nanotechnol. 2017, 12, 360–367. 10.1038/nnano.2016.267. 27992411

[ref17] Lucas F. L. R.; Versloot R. C. A.; Yakovlieva L.; Walvoort M. T.; Maglia G. Protein Identification by Nanopore Peptide Profiling. Nat. Commun. 2021, 12, 1–9. 10.1038/s41467-021-26046-9. 34608150 PMC8490355

[ref18] Wu H.-C.; Astier Y.; Maglia G.; Mikhailova E.; Bayley H. Protein Nanopores with Covalently Attached Molecular Adapters. J. Am. Chem. Soc. 2007, 129, 16142–16148. 10.1021/ja0761840. 18047341

[ref19] Li X.; Ying Y.-L.; Fu X.-X.; Wan Y.-J.; Long Y.-T. Single-Molecule Frequency Fingerprint for Ion Interaction Networks in a Confined Nanopore. Angew. Chem., Int. Ed. 2021, 60, 24582–24587. 10.1002/anie.202108226. 34390607

[ref20] Lucas F. L. R.; Willems K.; Tadema M. J.; Tych K. M.; Maglia G.; Wloka C. Unbiased Data Analysis for the Parameterization of Fast Translocation Events through Nanopores. ACS Omega 2022, 26040 10.1021/acsomega.2c00871. 35936408 PMC9352258

[ref21] Forstater J. H.; Briggs K.; Robertson J. W.; Ettedgui J.; Marie-Rose O.; Vaz C.; Kasianowicz J. J.; Tabard-Cossa V.; Balijepalli A. Mosaic: A Modular Single-Molecule Analysis Interface for Decoding Multistate Nanopore Data. Anal. Chem. 2016, 88, 11900–11907. 10.1021/acs.analchem.6b03725. 27797501 PMC5516951

[ref22] Plesa C.; Dekker C. Data Analysis Methods for Solid-State Nanopores. Nanotechnology 2015, 26, 084003 10.1088/0957-4484/26/8/084003. 25648179

[ref23] Wen C.; Dematties D.; Zhang S.-L. A Guide to Signal Processing Algorithms for Nanopore Sensors. ACS Sens. 2021, 6, 3536–3555. 10.1021/acssensors.1c01618. 34601866 PMC8546757

[ref24] Wang H.-F.; Huang F.; Gu Z.; Hu Z.-L.; Ying Y.-L.; Yan B.-Y.; Long Y.-T. Real-Time Event Recognition and Analysis System for Nanopore Study. Chin. J. Anal. Chem. 2018, 46, 843–850. 10.1016/S1872-2040(18)61090-4.

[ref25] Raillon C.; Granjon P.; Graf M.; Steinbock L.; Radenovic A. Fast and Automatic Processing of Multi-Level Events in Nanopore Translocation Experiments. Nanoscale 2012, 4, 4916–4924. 10.1039/c2nr30951c. 22786690

[ref26] Balijepalli A.; Ettedgui J.; Cornio A. T.; Robertson J. W.; Cheung K. P.; Kasianowicz J. J.; Vaz C. Quantifying Short-Lived Events in Multistate Ionic Current Measurements. ACS Nano 2014, 8, 1547–1553. 10.1021/nn405761y. 24397836 PMC3943493

[ref27] Gu Z.; Ying Y.-L.; Cao C.; He P.; Long Y.-T. Accurate Data Process for Nanopore Analysis. Anal. Chem. 2015, 87, 907–913. 10.1021/ac5028758. 25514172

[ref28] Zhang J.; Liu X.; Ying Y.-L.; Gu Z.; Meng F.-N.; Long Y.-T. High-Bandwidth Nanopore Data Analysis by Using a Modified Hidden Markov Model. Nanoscale 2017, 9, 3458–3465. 10.1039/C6NR09135K. 28232981

[ref29] Dematties D.; Wen C.; Pérez M. D.; Zhou D.; Zhang S.-L. Deep Learning of Nanopore Sensing Signals Using a Bi-Path Network. ACS Nano 2021, 15, 14419–14429. 10.1021/acsnano.1c03842. 34583465 PMC8482760

[ref30] Devlin J.; Chang M.-W.; Lee K.; Toutanova K. BERT: Pre-training of Deep Bidirectional Transformers for Language Understanding. 2019, 1810.04805, arXiv, https://arxiv.org/abs/1810.04805 (accessed April 17, 2022).

[ref31] Brown T. B.; Mann B.; Ryder N.; Subbiah M.; Kaplan J.; Dhariwal P.; Neelakantan A.; Shyam P.; Sastry G.; Askell A.; Agarwal S.; Herbert-Voss A.; Krueger G.; Henighan T.; Child R.; Ramesh A.; Ziegler D. M.; Wu J.; Winter C.; Hesse C.; Chen M.; Sigler E.; Litwin M.; Gray S.; Chess B.; Clark J.; Berner C.; McCandlish S.; Radford A.; Sutskever I.; Amodei D. Language Models Are Few-Shot Learners. 2020, 2005.14165, arXiv, https://arxiv.org/abs/2005.14165 (accessed April 17, 2022).

[ref32] Jumper J.; Evans R.; Pritzel A.; Green T.; Figurnov M.; Ronneberger O.; Tunyasuvunakool K.; Bates R.; Zidek A.; Potapenko A.; Bridgland A.; Meyer C.; Kohl S. A. A.; Ballard A. J.; Cowie A.; Romera-Paredes B.; Nikolov S.; Jain R.; Adler J.; Back T.; Petersen S.; Reiman D.; Clancy E.; Zielinski M.; Steinegger M.; Pacholska M.; Berghammer T.; Bodenstein S.; Silver D.; Vinyals O.; Senior A. W.; Kavukcuoglu K.; Kohli P.; Hassabis D. Highly Accurate Protein Structure Prediction with AlphaFold. Nature 2021, 596, 583–589. 10.1038/s41586-021-03819-2. 34265844 PMC8371605

[ref33] Dosovitskiy A.; Beyer L.; Kolesnikov A.; Weissenborn D.; Zhai X.; Unterthiner T.; Dehghani M.; Minderer M.; Heigold G.; Gelly S.; Uszkoreit J.; Houlsby N. An Image Is Worth 16×16 Words: Transformers for Image Recognition at Scale. 2021, 2010.11929, arXiv, https://arxiv.org/abs/2010.11929 (accessed April 17, 2022).

[ref34] Carion N.; Massa F.; Synnaeve G.; Usunier N.; Kirillov A.; Zagoruyko S. End-to-End Object Detection with Transformers. 2020, 2005.12872, arXiv, https://arxiv.org/abs/2005.12872 (accessed April 17, 2022).

[ref35] Alzubaidi L.; Zhang J.; Humaidi A. J.; Al-Dujaili A.; Duan Y.; Al-Shamma O.; Santamarıa J.; Fadhel M. A.; Al-Amidie M.; Farhan L. Review of Deep Learning: Concepts, CNN Architectures, Challenges, Applications, Future Directions. J. Big Data 2021, 8, 53 10.1186/s40537-021-00444-8. 33816053 PMC8010506

[ref36] Lin T.-Y.; Maire M.; Belongie S.; Bourdev L.; Girshick R.; Hays J.; Perona P.; Ramanan D.; Zitnick C. L.; Dollár P. Microsoft COCO - Common Objects in Context. 2014, 1405.0312, arXiv, https://arxiv.org/abs/1405.0312 (accessed April 17, 2022).

[ref37] Girshick R.; Donahue J.; Darrell T.; Malik J. Rich Feature Hierarchies for Accurate Object Detection and Semantic Segmentation. 2014, 1311.2524, arXiv, https://arxiv.org/abs/1311.2524 (accessed April 17, 2022).

[ref38] Tan M.; Pang R.; Le Q. V. EfficientDet: Scalable and Efficient Object Detection. 2020, 1911.09070, arXiv, https://arxiv.org/abs/1911.09070 (accessed April 17, 2022).

[ref39] Redmon J.; Divvala S.; Girshick R.; Farhadi A. You Only Look Once: Unified, Real-Time Object Detection. 2016, 1506.02640, arXiv, https://arxiv.org/abs/1506.02640 (accessed April 17, 2022).

[ref40] Xue L.; Yamazaki H.; Ren R.; Wanunu M.; Ivanov A. P.; Edel J. B. Solid-State Nanopore Sensors. Nat. Rev. Mater. 2020, 5, 931–951. 10.1038/s41578-020-0229-6.

[ref41] Wen C.; Zhang S.-L. Fundamentals and Potentials of Solid-State Nanopores: A Review. J. Phys. D: Appl. Phys. 2020, 54, 023001.

[ref42] Wen C.; Zeng S.; Arstila K.; Sajavaara T.; Zhu Y.; Zhang Z.; Zhang S.-L. Generalized Noise Study of Solid-State Nanopores at Low Frequencies. ACS Sens. 2017, 2, 300–307. 10.1021/acssensors.6b00826. 28723146

[ref43] Fragasso A.; Schmid S.; Dekker C. Comparing Current Noise in Biological and Solid-State Nanopores. ACS Nano 2020, 14, 1338–1349. 10.1021/acsnano.9b09353. 32049492 PMC7045697

[ref44] Zeng S.; Wen C.; Solomon P.; Zhang S.-L.; Zhang Z. Rectification of Protein Translocation in Truncated Pyramidal Nanopores. Nat. Nanotechnol. 2019, 14, 1056–1062. 10.1038/s41565-019-0549-0. 31591525

[ref45] Plesa C.; Kowalczyk S. W.; Zinsmeester R.; Grosberg A. Y.; Rabin Y.; Dekker C. Fast Translocation of Proteins through Solid State Nanopores. Nano Lett. 2013, 13, 658–663. 10.1021/nl3042678. 23343345 PMC4151282

[ref46] Pham N. H.; Yao Y.; Wen C.; Li S.; Zeng S.; Nyberg T.; Tran T. T.; Primetzhofer D.; Zhang Z.; Zhang S.-L. Self-Limited Formation of Bowl-Shaped Nanopores for Directional DNA Translocation. ACS Nano 2021, 15, 17938–17946. 10.1021/acsnano.1c06321. PMC8613906 34762404

[ref47] English B. P.; Min W.; Van Oijen A. M.; Lee K. T.; Luo G.; Sun H.; Cherayil B. J.; Kou S.; Xie X. S. Ever-Fluctuating Single Enzyme Molecules: Michaelis-Menten Equation Revisited. Nat. Chem. Biol. 2006, 2, 87–94. 10.1038/nchembio759. 16415859

[ref48] Vaswani A.; Shazeer N.; Parmar N.; Uszkoreit J.; Jones L.; Gomez A.N.; Kaiser L.; Polosukhin I. Attention Is All you Need. 2017, 1706.03762, arXiv, https://arxiv.org/abs/1706.03762 (accessed April 17, 2022).

[ref49] Dematties D.; Wen C.; Pérez M.; Zhou D.; Zhang S.-L. Nanopore Translocation Signal. 2021. 10.5281/zenodo.5013856 (accessed April 17, 2022).

