# Supplementary material for: A Generalized Transformer-Based Pulse Detection Algorithm
Source: ACS Sens. 2022 Aug 30;7(9):2710–20. doi: 10.1021/acssensors.2c01218 (PMC9513795; doi:10.1021/acssensors.2c01218)
Supplement: Supplementary file 1 — se2c01218_si_001.pdf [file se2c01218_si_001.pdf]

Supporting information

A Generalized Transformer-Based Pulse Detection Algorithm

Dario Dematties,[1,2†] Chenyu Wen,[3,4†] and Shi-Li Zhang[5*]
1 Northwestern Argonne Institute of Science and Engineering, Northwestern University, 2205 Tech Drive Suite 1-160, Evanston, 60208, IL, USA.
2 Mathematics and Computer Science Division, Argonne National Laboratory, 9700 S. Cass Avenue, Lemont, 60439, IL, USA.
3 NanoDynamicsLab, Laboratory of Biophysics, Wageningen University, Stippeneng 4, Wageningen, 6708 WE, The Netherlands.
4 Department of Bionanoscience, Kavli Institute of Nanoscience, Delft University of Technology, Van der Maasweg 9, Delft, 2629 HZ, The Netherlands.
5 Department of Electrical Engineering, Uppsala University, Lägerhyddsvägen 1, 752 37, Uppsala, SE-751 03, Uppsala, Sweden.
*Corresponding author(s). E-mail(s): shili.zhang@angstrom.uu.se;
†These authors contributed equally to this work.

Table of Contents:

Note 1: About mean average precision and our adaptation
Note 2: Detailed results from the PETR
- 2.1. Examples of spike recognition in SNR=4 dataset
- 2.2. Artificially generated traces. SNR = 4, 2, 1, 0.5, and 0.25
- 2.3. Interpolated data. SNR = 4
- 2.4. Using IoU on SNR = 4 data

Note 3: Comparison with B-Net and traditional method
Note 4: DBC and ADEPT

Supporting Note 1: About mean average precision and our adaptation

There are cases for which the Pulse dEtection TRansformer (PETR) algorithm produces detections with high Intersection on Union (IoU) values, as showed in Figs. S1(a) and (b). Even when PETR does not produce a high IoU value for cases shown in Figs. S1(c) and (d), the detection duration is well predicted and pulses can be easily identified and individualized per detection. Consequently, basing the detection performance of PETR on computing mAP using IoU is not convenient given the characteristics of the task. Other alternatives could reflect more accurately the performance of the algorithm. Therefore, we used an adapted performance metric for the evaluation.

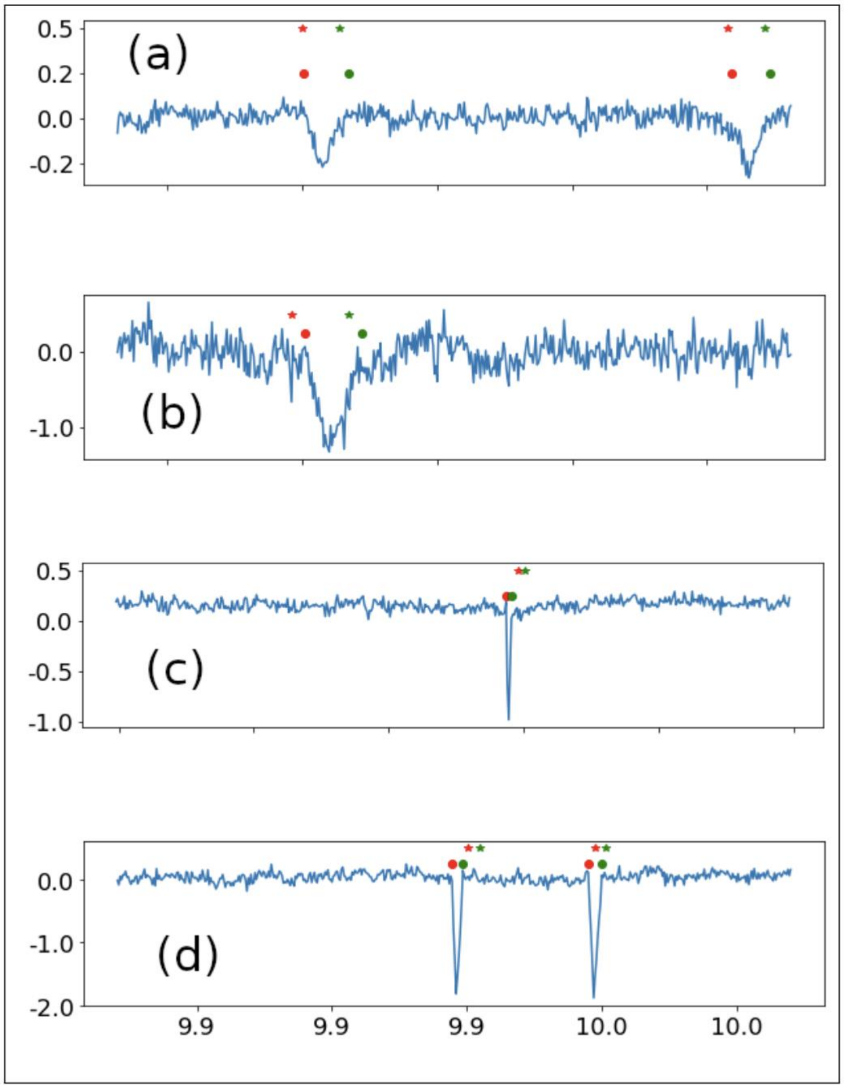

Fig. S1. Four examples of detections produced by PETR on different trace windows. (a) and (b) present two examples on SNR of 1, while in (c) and (d) we have two examples on SNR of 4. Stars represent the predictions produced by PETR while dots are the ground truths.

The adaptation implemented to the metric in order to evaluate PETR is shown in Fig. S2. The classical IoU used to compute mAP can be seen in Fig. S2(b). Basically, pulses are pinpointed by computing the value of the intersection between the prediction and the ground truth and then dividing such a value by the union

between both. This metric returns an idea of how well the prediction represents the ground truth. If a value of IoU equal to 0.5 is considered as threshold, it means that only predictions with IoUs above such a threshold will be considered as true positives.

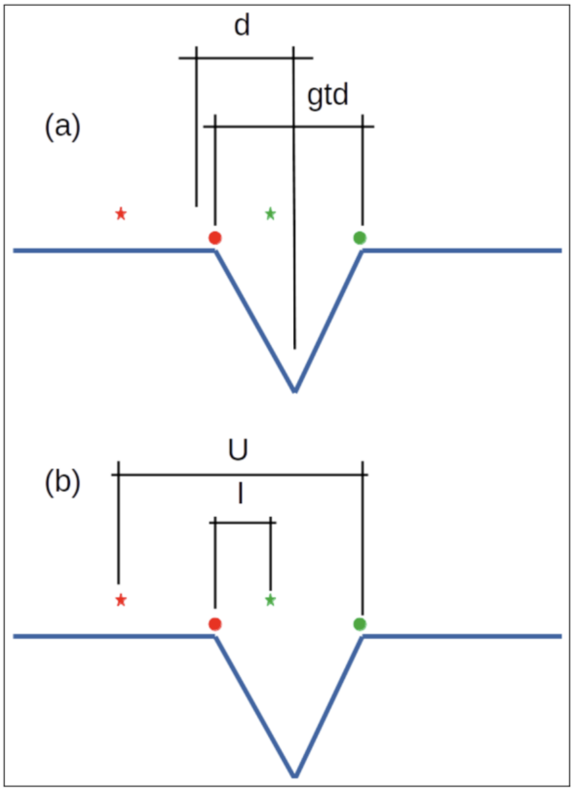

Fig. S2. Illustration of the difference between the classical IoU computation (b) and our adaptation computing the relative distance between the prediction and the ground truth (a). Stars represent the predictions produced by PETR while dots are the ground truths.

Yet, such a metric neglects many good detections produced by PETR as the ones shown in Figs. S1 (c and d). Excepting for the pulse around 10.0 in Fig. S1(d), the IoU value for such cases is 0 and their predictions become neglected even when they are easily detectable. To mitigate, the metric applying the one shown in Fig. S2(a) is adapted. Instead of IoU, the distance between the mid-points of the prediction and the ground truth is computed. It is then divided by the duration of the ground truth. This new metric allows us to associate predictions with labels even in situations with their IoU being 0. For instance, the relative distance between the prediction and the ground truth in Fig. S1(c) is greater than 100%. Using a threshold of 400% could be enough to catch such a pulse with the prediction produced by PETR. This enables us to consider such a prediction as a true positive and allows us to analyze the surroundings of the prediction where the real pulse locates.

Supporting Note 2: Detailed results from PETR

2.1. Examples of spike recognition in SNR=4 dataset

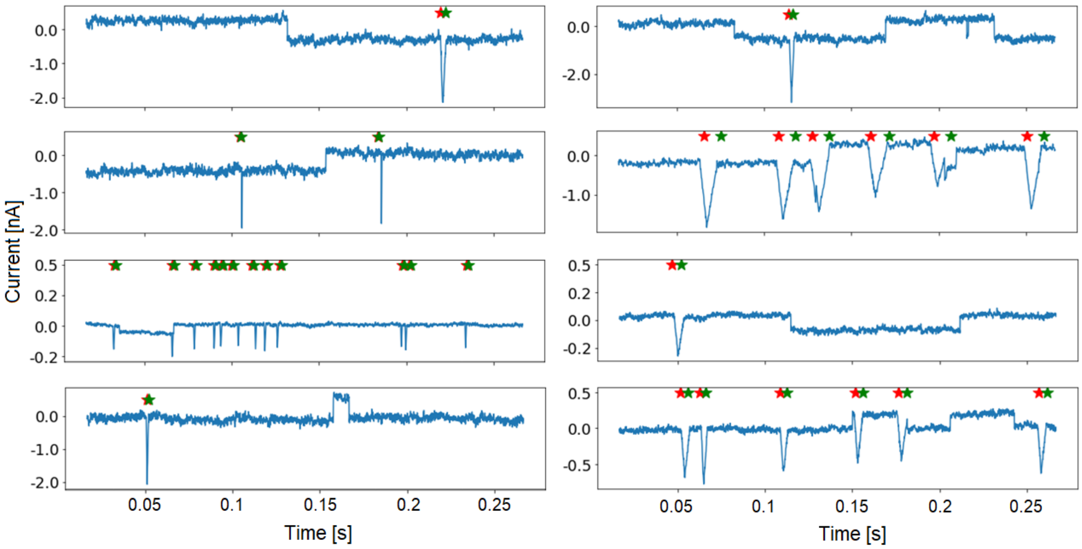

Fig. S3. Examples of recognition pulses by PETR on the dataset of SNR=4. The red and green starts mark the start and end time points of pulses predicted by PETR. The baseline shows significant fluctuations with sudden jumps, while the PETR is immune to them.

2.2. Artificially generated traces. SNR = 4, 2, 1, 0.5, 0.25

2.2.1. mAP

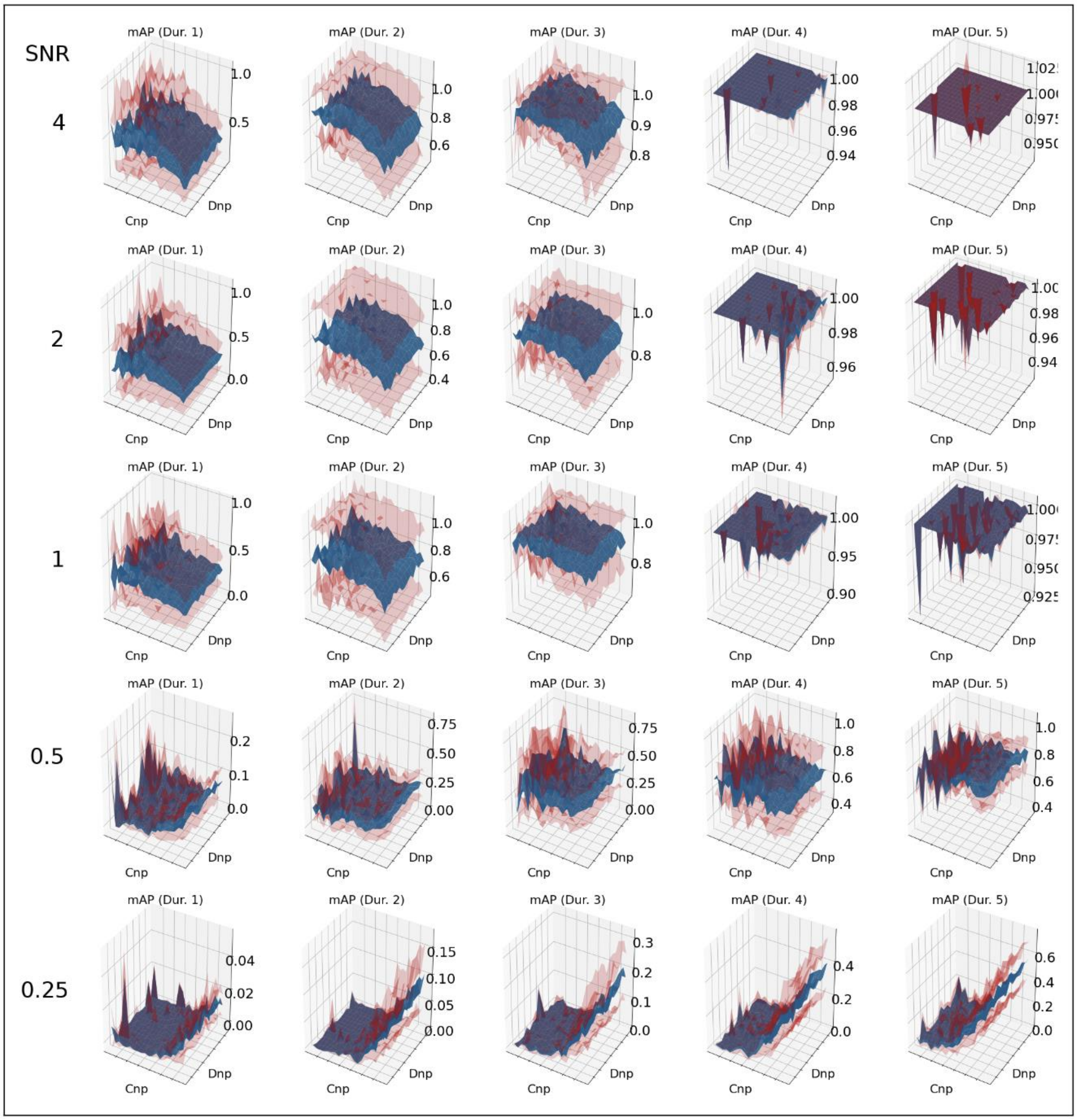

Fig. S4. mAP computed using relative distance instead of IoU for different SNRs and Durations. Blue surfaces represent mean values while translucent red ones represent Standard Deviations (STDs). The domain in each surface is the cartesian between concentration of analytes (Cnps) and diameter of nanopores (Dnps).

2.2.2. Coverage

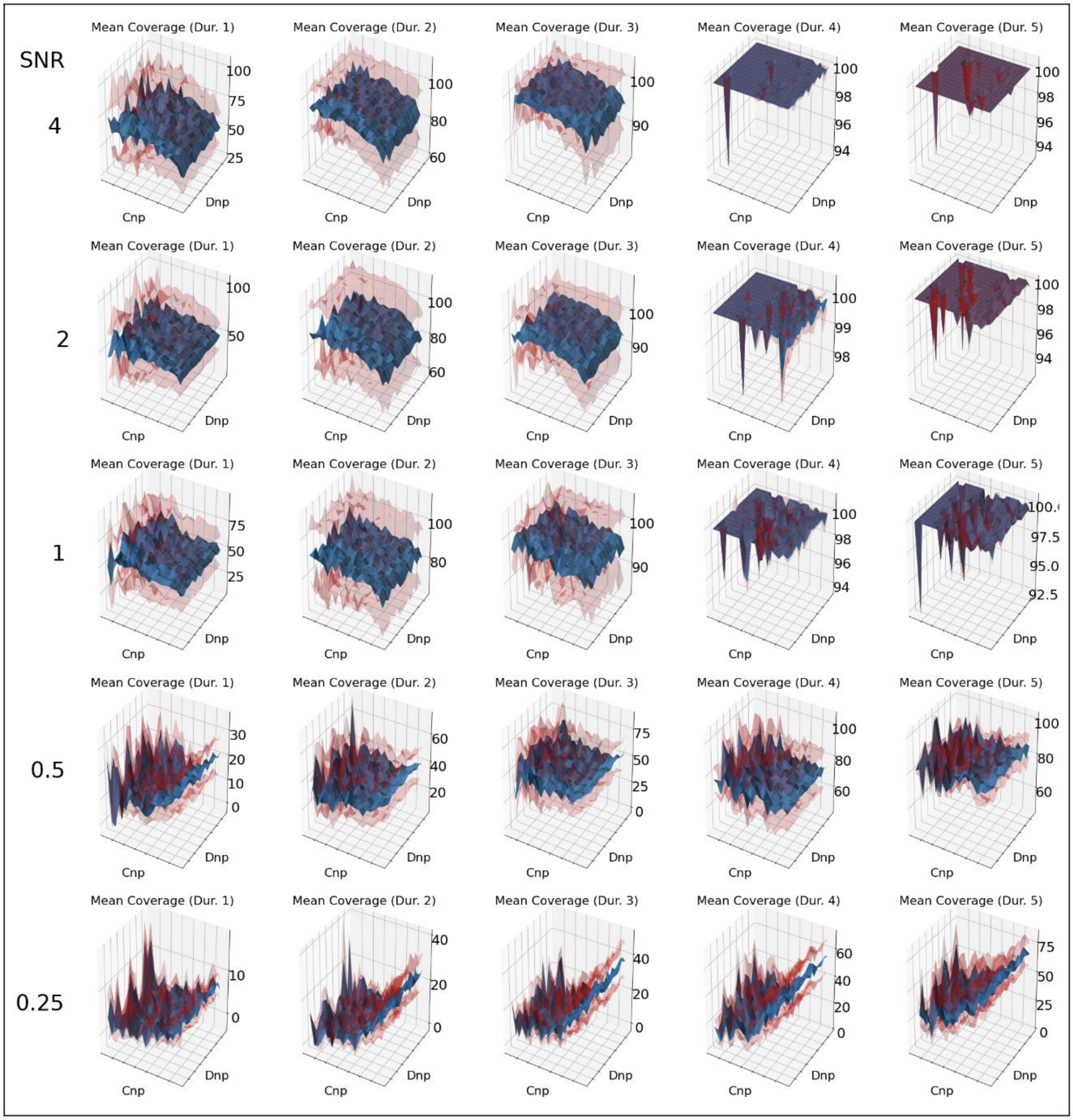

Fig. S5. Coverage is the total number of true positives predicted by PETR divided by the total number of ground truth segments. Blue surfaces represent mean values while translucent red ones represent STDs. The domain in each surface is the cartesian between Cnps and Dnps.

2.2.3. Duration error

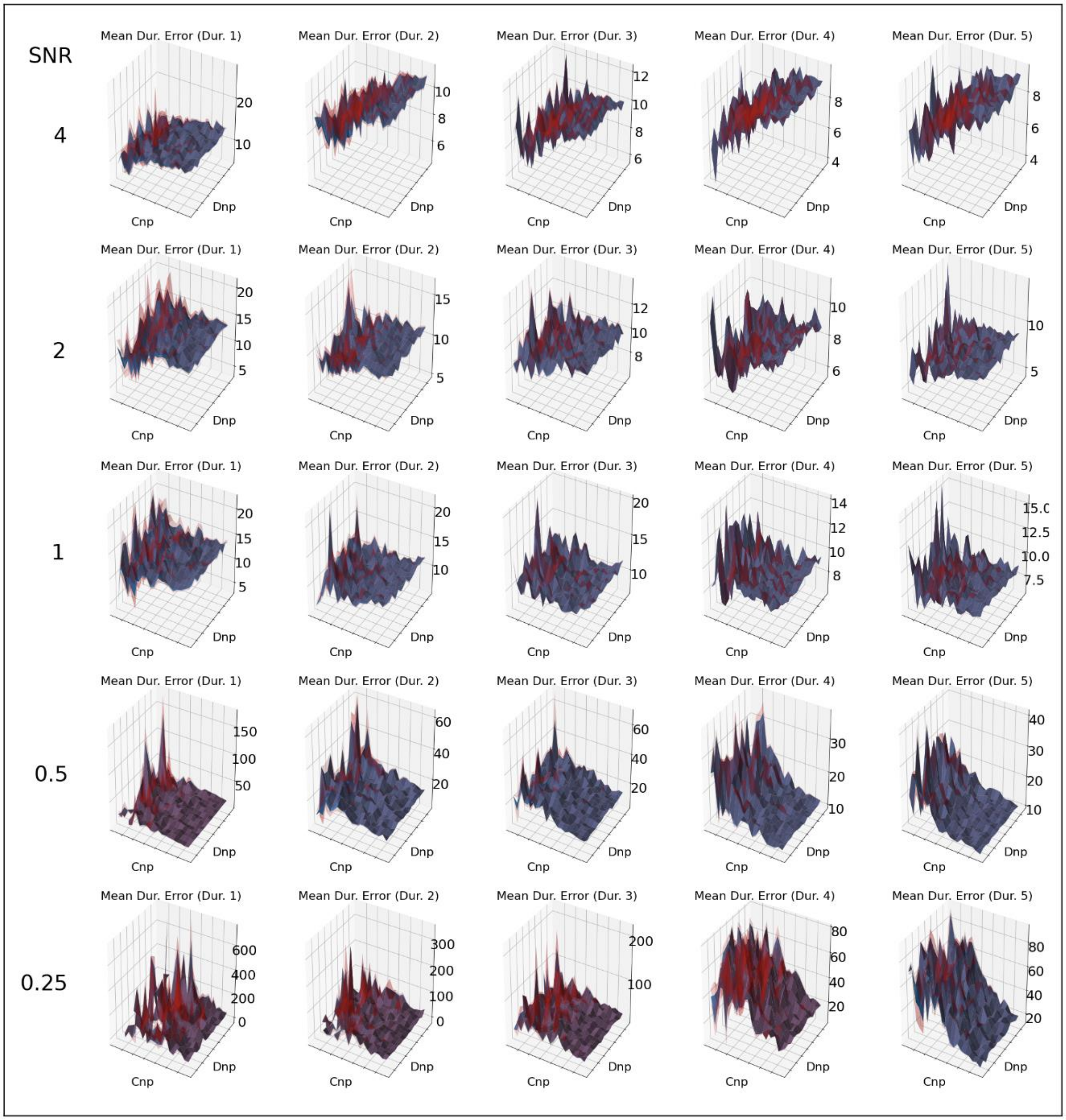

Fig. S6. Relative duration errors for different SNRs and Durations. Blue surfaces represent mean values while translucent red ones represent STDs. The domain in each surface is the cartesian between Cnps and Dnps.

2.2.4. Start and end time error

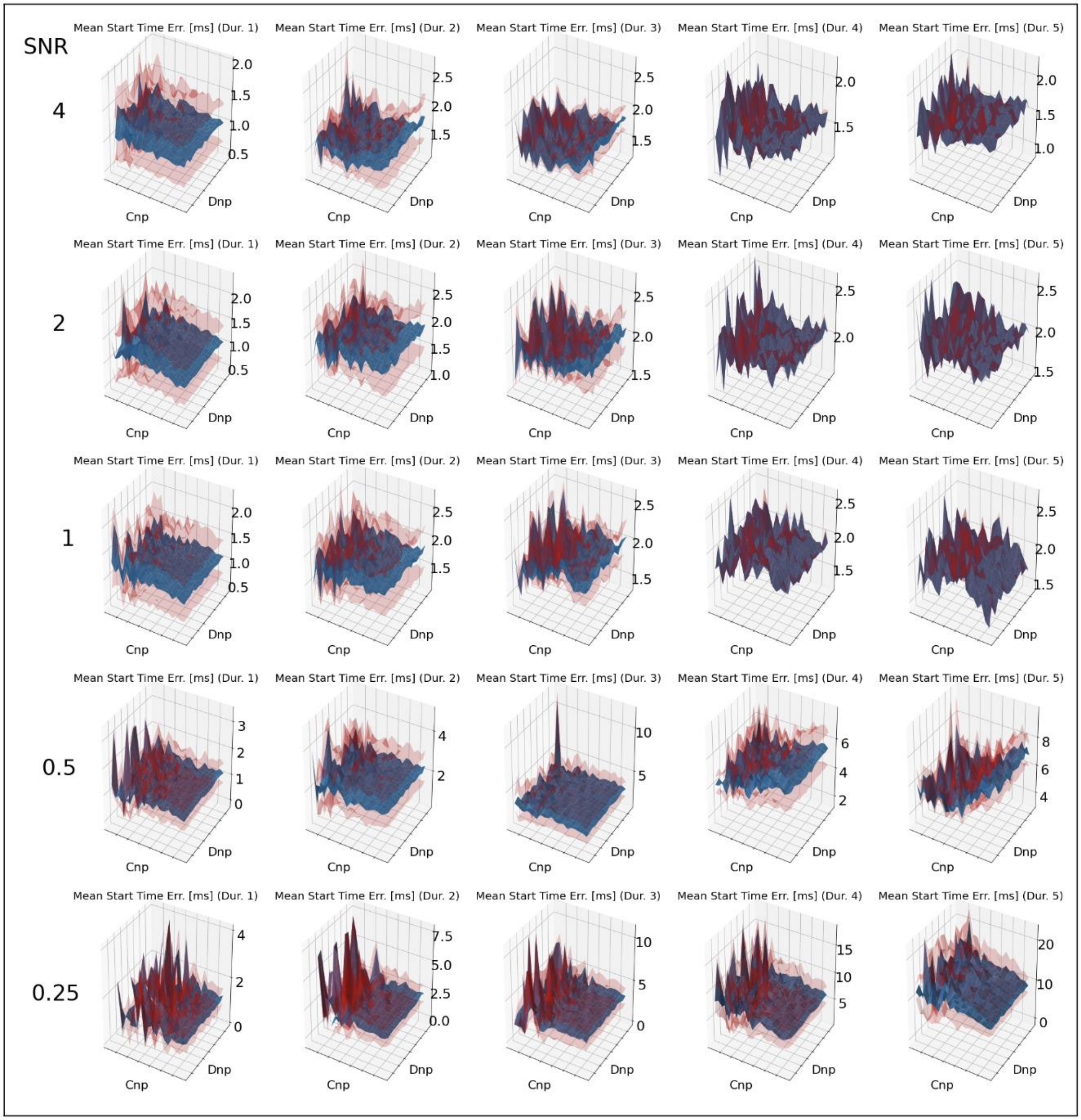

Fig. S7. Mean Start Time errors for different SNRs and Durations. Blue surfaces represent mean values while translucent red ones represent STDs. The domain in each surface is the cartesian between Cnps and Dnps.

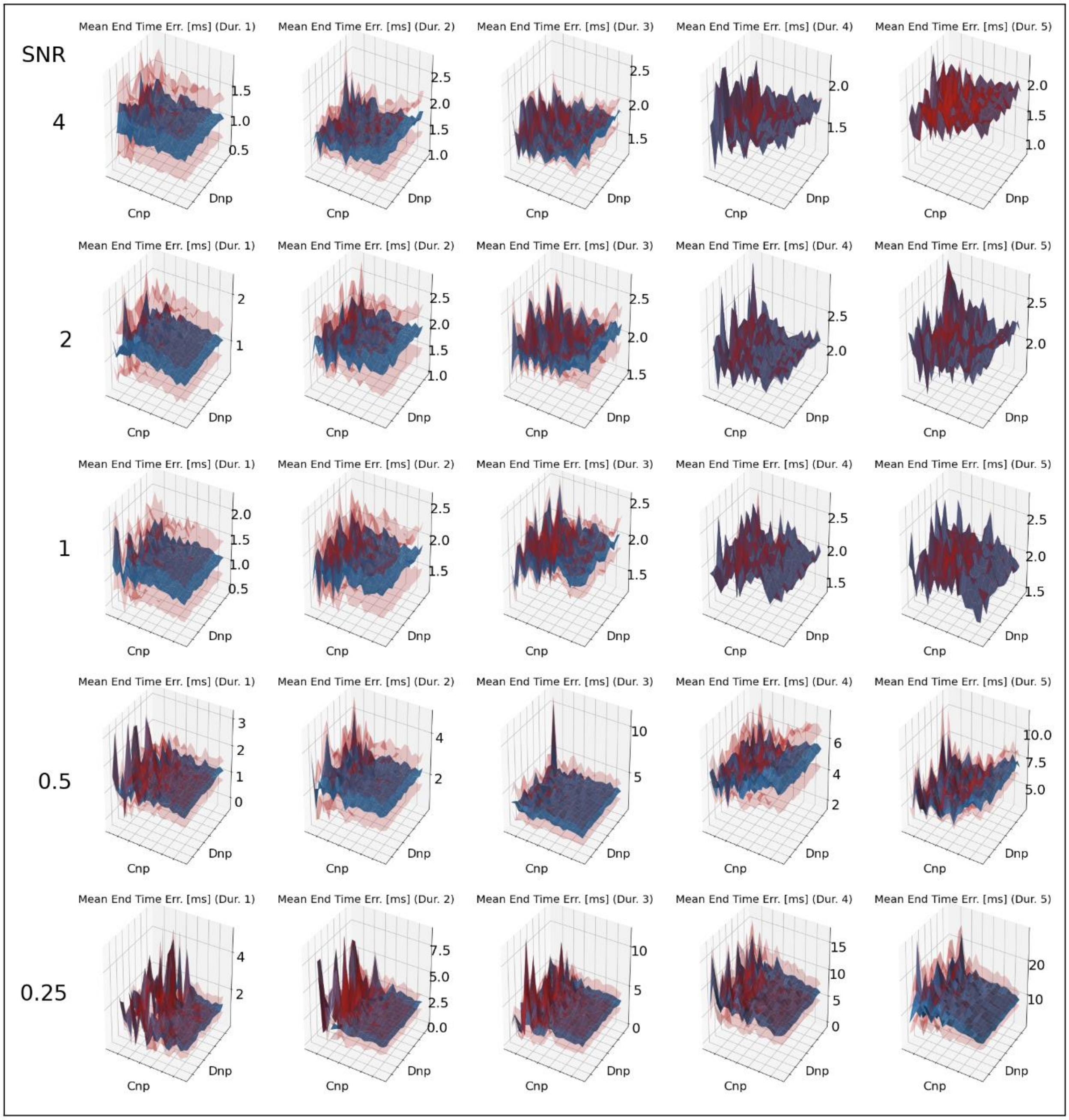

Fig. S8. Mean End Time errors for different SNRs and Durations. Blue surfaces represent mean values while translucent red ones represent STDs. The domain in each surface is the cartesian between Cnps and Dnps.

2.2.5. Detection performance summary

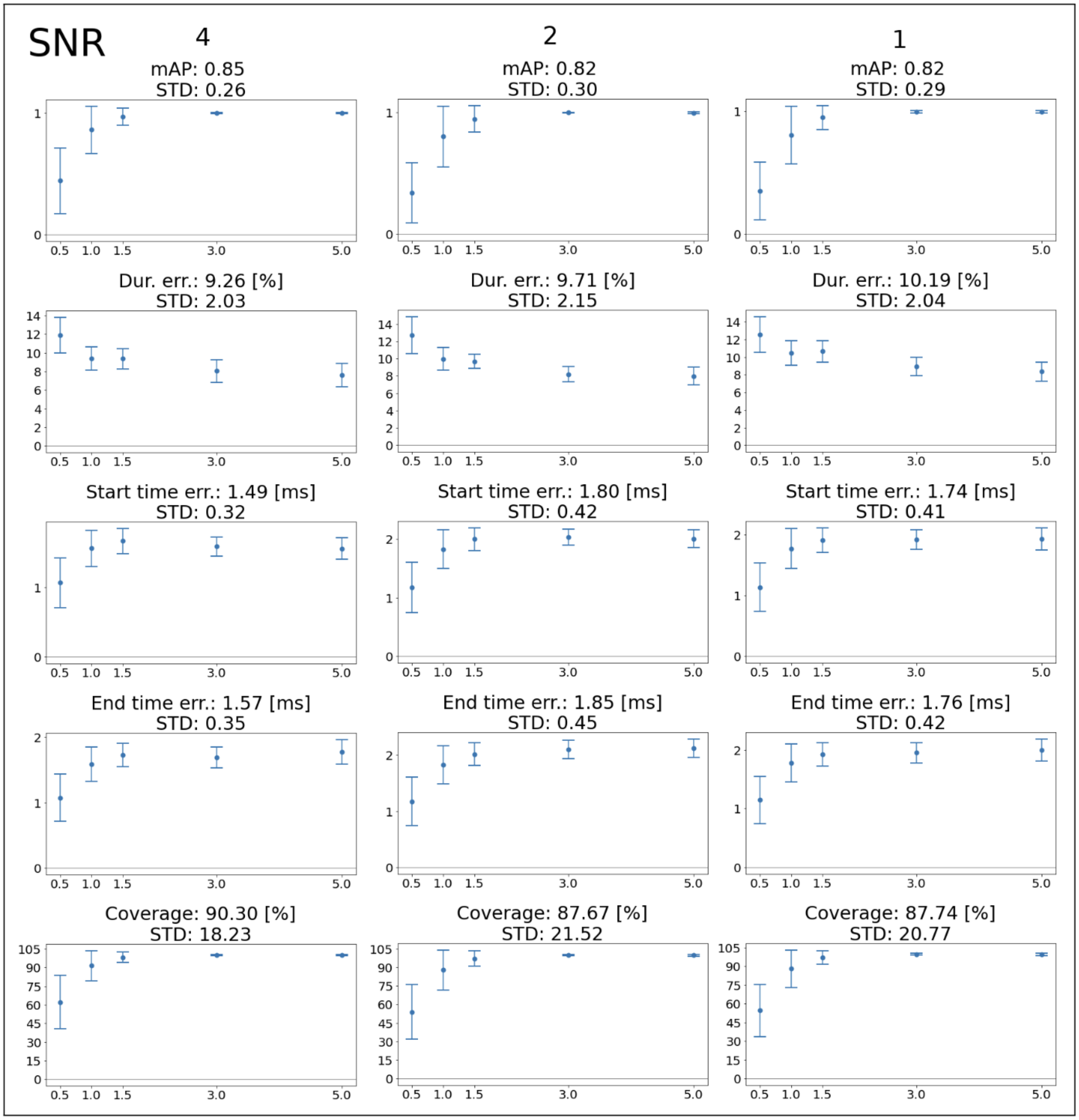

Fig. S9. Detection performance summary for different levels of SNR (4, 2, and 1). Each column represents a different level of SNR. The first row shows our adapted mAP. The second row corresponds to the Duration Error. The third and fourth rows correspond, respectively, to the Start and End time errors of the predicted time marks. Finally, the fifth row gives the coverage.

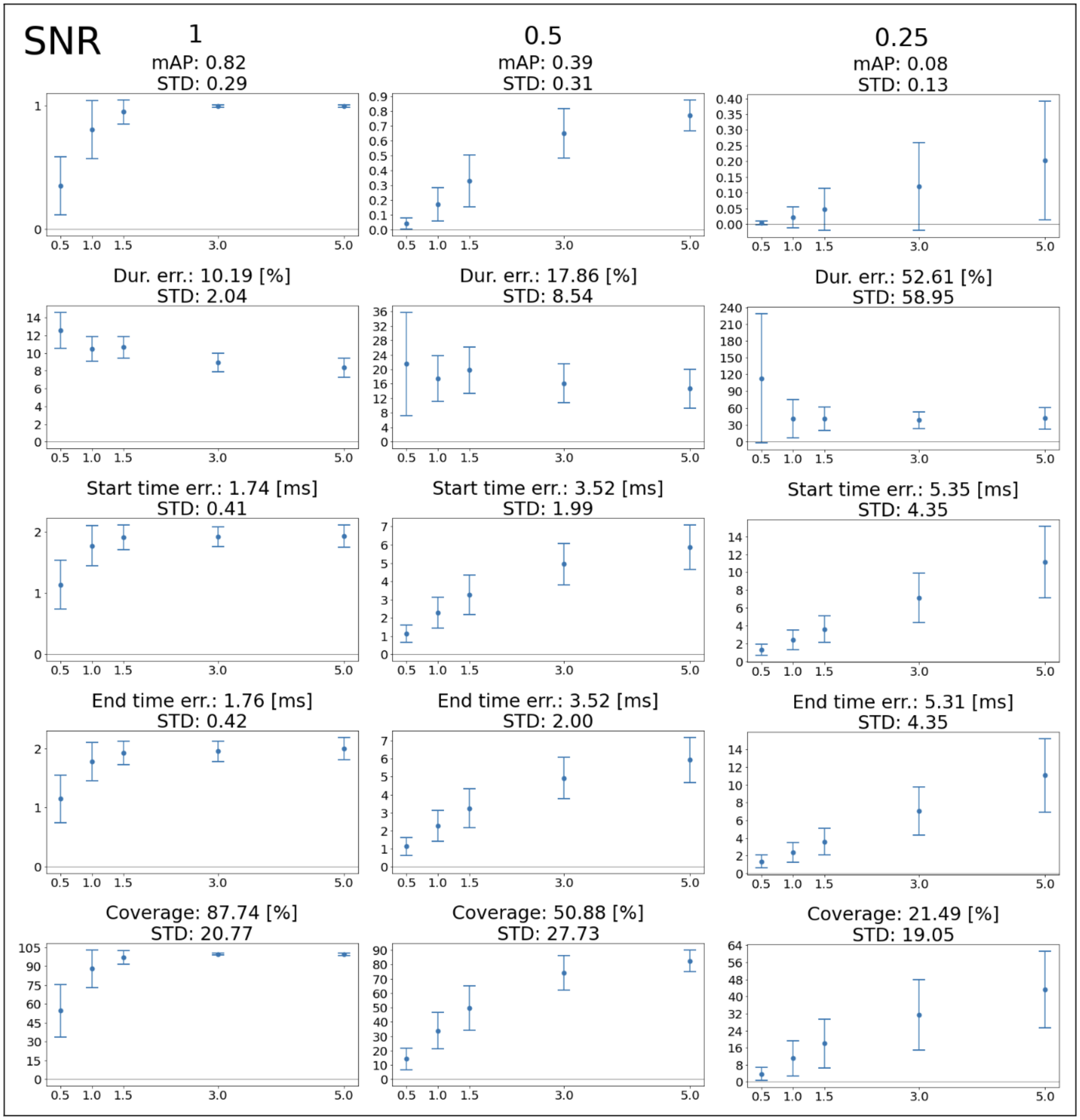

Fig. S10. Detection performance summary for different levels of SNR (1, 0.5, and 0.25). Each column represents a different level of SNR. The first row shows our adapted mAP. The second row corresponds to the Duration Error. The third and fourth rows correspond to the Start and End time errors of the predicted time marks. Finally, the fifth row gives the coverage.

2.3. Interpolated data. SNR = 4

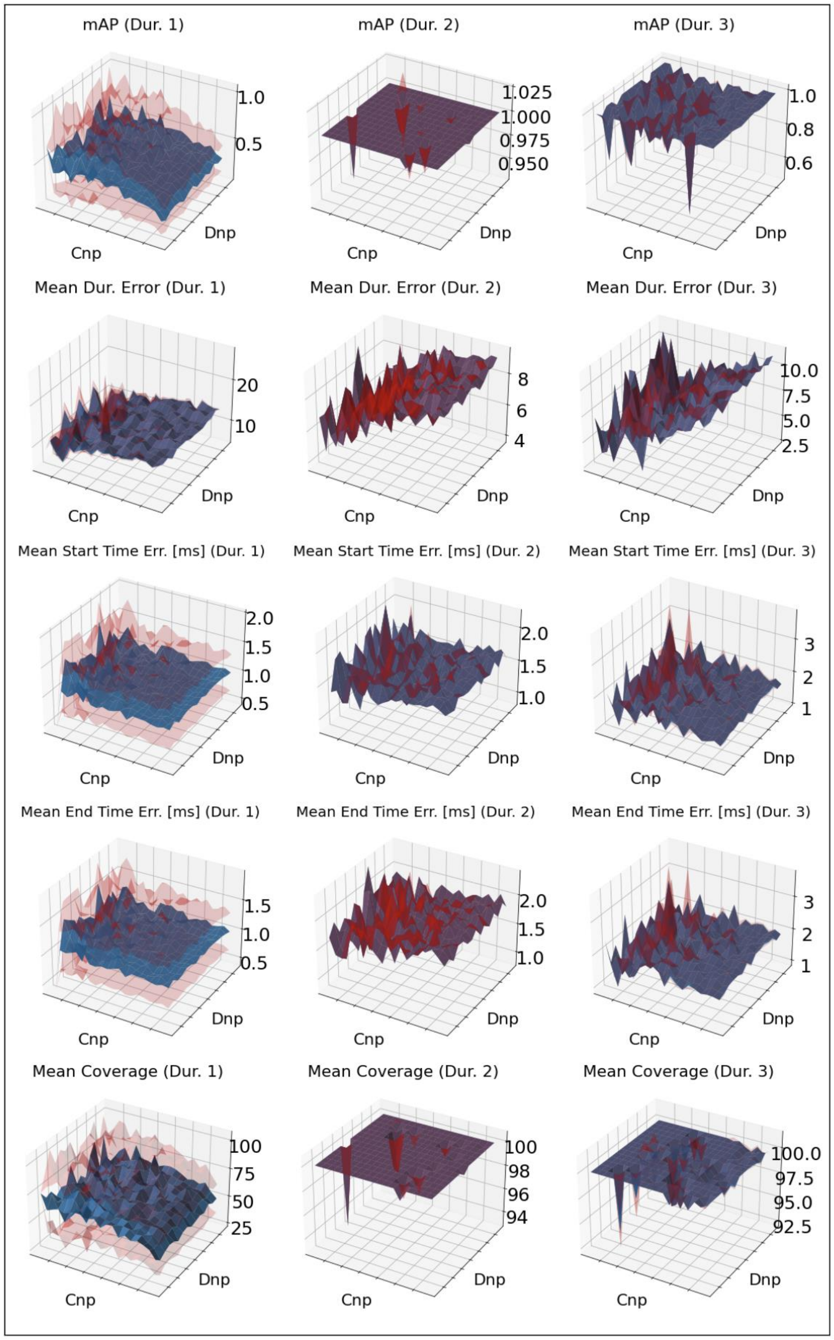

Fig. S11. Data Interpolation. This figure illustrates how the interpolation of the traces can improve the detection performance to levels similar to the ones obtained for larger translocation durations. First row shows our adapted mAP. Second row shows the Duration Error. Third and fourth rows show, respectively, the start and end time errors. Finally, the fifth row shows the Coverage. First column (Dur 1) corresponds to traces with translocations whose duration is 0.5 ms. Second column (Dur 2) corresponds to traces with translocations whose duration is 5 ms. Finally, the third column (Dur 3) corresponds to traces with translocations whose duration is 0.5 ms but such traces are interpolated with additional points in order to achieve an apparent duration of 5 ms.

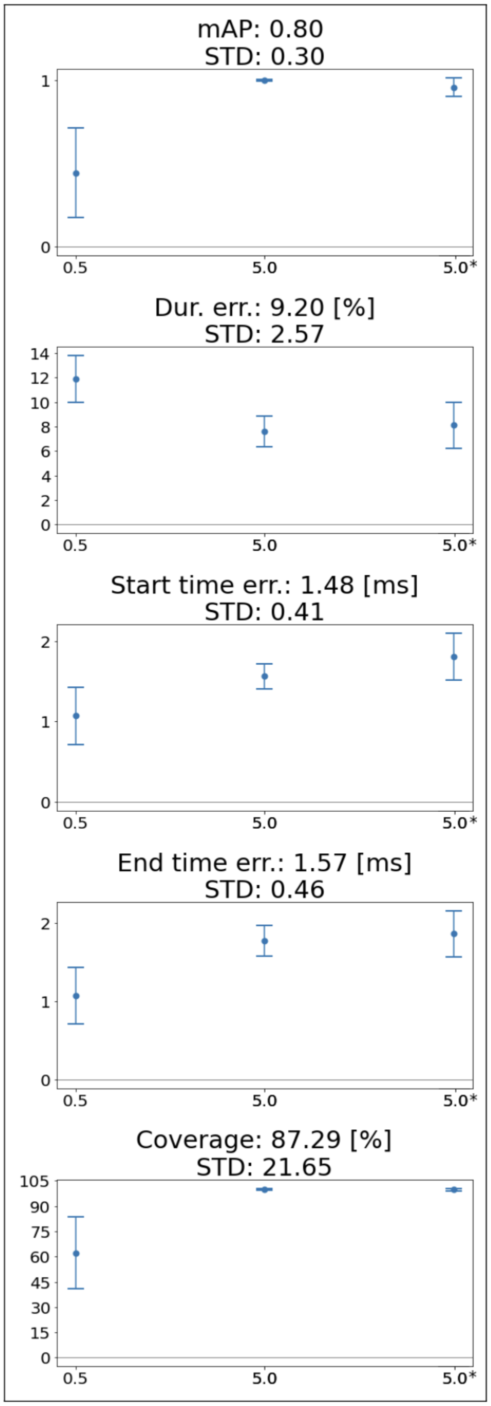

Fig. S12. General comparison of the pulse extraction performance among short duration dataset, long duration dataset, and the extrapolated dataset. First row: adapted mAP. Second row: duration Error. Third and fourth rows: the start and end time errors. Fifth row: Coverage. The value above each figure shows the average and standard deviation of the three datasets. First value (0.5), corresponds to traces with translocations whose duration is 0.5 ms. Second value (5.0), corresponds to traces with translocations whose duration is 5 ms. Finally, third value (5.0*), corresponds to traces with translocations whose duration is 0.5 ms but such traces are interpolated with additional points to achieve an apparent duration of 5 ms.

2.4. Using IoU on SNR = 4 dataset

Details of the performance measured by the standard mAP for different translocation durations can be seen in Fig. S13. By evaluating our model using the standard mAP, the obtained results still show a ≥ 0.3 mAP for 5 ms duration spikes, which is comparable to detectors used in other application scenarios [1–4].

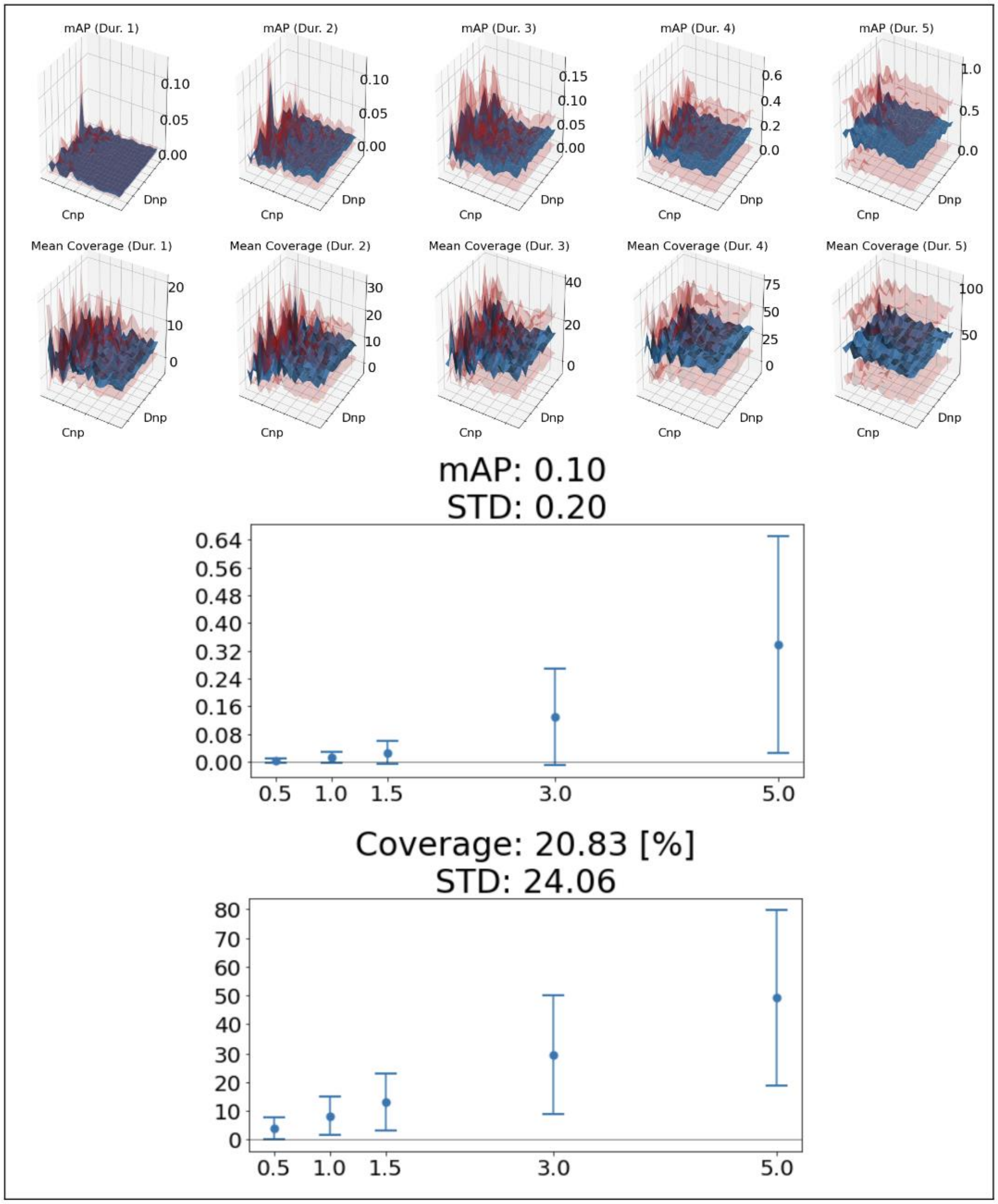

Fig. S13. mAP and Coverage values when using IoU instead of Relative Distance as in previous experiments. The first row of plots shows surfaces of mAP for different values of translocation duration. The second row is the same as the first one but for Coverage. Blue surfaces show the mean while transparent red ones show the STD. The upper and lower diagrams show mAP and Coverage, respectively, as mean values and STD presented as error bars, for different durations.

Supporting Note 3: Comparison with B-Net and traditional method

The traditional method to detect pulses is based on an amplitude threshold referring to the baseline [5]. If the amplitude of a fluctuation in the signal trace surpasses this threshold, it is recognized as a pulse. Here, the threshold-based algorithm is implemented by a MATLAB program to locate the translocation spikes in current traces. In the program, the function *findpeaks* is adopted with the *MinPeakProminence* method [6, 7]. An amplitude threshold is defined by the user regarding the Root-Mean-Square (RMS) of the background noise level. This threshold is tuned from 4 to 25 multiples of the background noise RMS to demonstrate the dependence of the results on the threshold selection.

The spike duration and frequency of λ-DNA and streptavidin translocation from traditional algorithm and PETR are compared in Fig. S14. The average values of the spike duration and frequency from the traditional algorithm are dependent on the selection of threshold amplitude, as shown by the deviations among the dot-on lines in the respective figures. “n” after “th” (for threshold) in the legend denotes a specific threshold level, measured by the number of multiples of the peak-to-peak value of the background noise. The feature extracted by the traditional algorithm are highly dependent on the selection of amplitude threshold, indicating the subjectivity of the threshold-based algorithm.

In the traditional method, different thresholds for distinguishing spikes from the background noise fluctuation assigned by the user give totally different statistical results on duration and frequency, not only the values, but also the trends with increasing bias voltage. Using PETR does not suffer from such subjectivity. Spike detection with PETR is based on the acquisition of spike features, referring to the properties of the background noise, by the neural network during the training process, instead of any user-defined parameter.

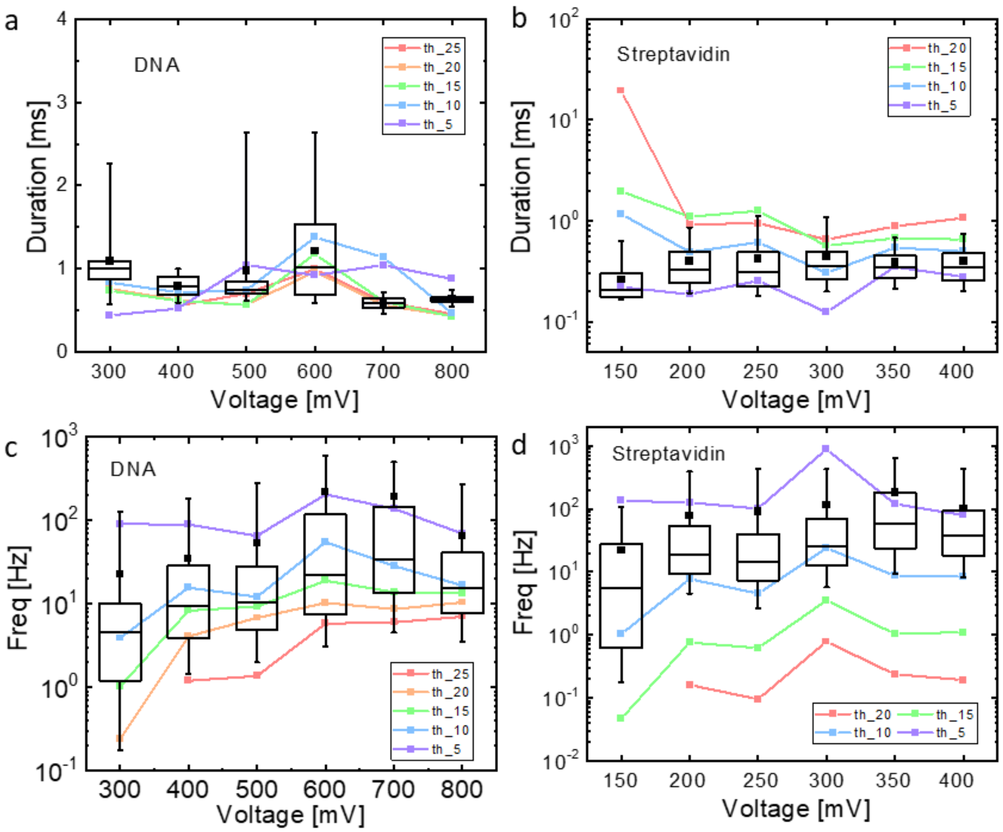

Fig. S14. Comparison of spike duration and frequency of λ-DNA and streptavidin translocation data extracted by PETR and the traditional method. The duration at different bias voltages for (a) λ-DNA and (b) streptavidin translocation data. The spike appearance frequency at different bias voltages for (c) λ-DNA and (d) streptavidin translocation. The box charts are from PETR and the colour dot-on-lines (average values) in each figure show the corresponding results from the traditional method with different thresholds referring to the background noise levels.

Supporting Note 4: DBC AND ADEPT

The spike segment outputs from PETR are processed using two published algorithms, Second-Order-Differential-Based Calibration (DBC) [8] and ADEPT [9]. The purpose these two algorithms share is to extract the features of each spikes, *i.e.*, the duration and amplitude of the spikes. The procedure of DBC is illustrated as follows using an example (Fig. S15).

1. The spike segment is fitted by an 8-order Fourier series to smooth out the noise, *i.e.*, orange curve in Fig. S15(a).

$$I(t) = a_0 + \sum_{n=1}^{8} a_n cos(n\omega t) + b_n sin(n\omega t) \quad \text{(S1)}$$

2. The second-order derivative of the smoothed spike segment is calculated, *i.e.*, pink curve in Fig. S15(b).
3. The valleys of the second-order derivative are founded, *i.e.*, green dots in Fig. S15(b), from which the first- and second-smallest two are registered as the start and end time points of the spike, respectively, *i.e.*, circled by the blue squares in Fig. S15(b). Then, the duration is the time interval between the start and end points.
4. The amplitude is calculated by averaging the area of spike waveform below the baseline during the translocation over the entire duration.

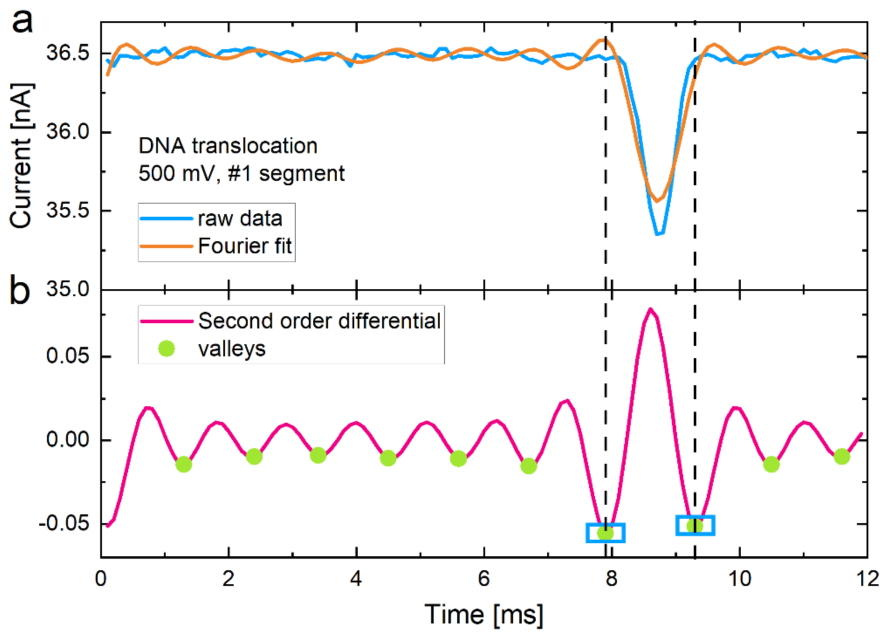

Fig. S15. Procedure of the DBC algorithm. (a) The 8 order Fourier series fitting. (b) Localization of the start and end time points of the translocation in the second derivative of the smoothed spike.

The procedure of ADEPT is illustrated as follows using an example (Fig. S16).

1. The baseline of the spike segment is subtracted, *i.e.*, Fig. S16(a).
2. The segment is fitted by a target function:

$$I(t) = a(-\mathcal{H}(t-ts)(1-\exp(-(t-ts)/\tau_1)) + \mathcal{H}(t-te)(1-\exp(-(t-te)/\tau_2))) \quad \text{(S2)}$$

Where, $H(t)$ is the heaviside function, marking the start time, *ts*, and end time, *te*, of the translocation. The two exponential functions describe the decrease and increase relaxation processes of the current caused by delay characteristics of the system triggered by the entering and exiting of the analyte during the translocation. $\tau1$ and $\tau2$ are the relaxation time constants for the current decrease and increase, respectively (Fig. S16(b)). 3. The duration can be calculated as the time difference between the start and end points, *i.e.*, $te - ts$. The amplitude is the fitting parameter a.

The extracted amplitude and duration of the spike segments by means of ADEPT and DBC are shown in Fig. S17. Three typical examples of the spike segments of λ-DNA and streptavidin are displayed in Fig. S17(a)-(b) with the start and end time points predicted by PETR marked by red and green stars, respectively. The spike amplitude increases with raising the bias voltage, which is reasonable since a higher voltage induces a larger ionic current through the nanopore. In general, DBC extracts a smaller spike amplitude compared to ADEPT. It relates to the flatting of the spike amplitude along the translocation time span, *i.e.*, averaging the changing current in the translocation duration. The spike duration of λ-DNA and streptavidin in Fig. S17(c)-(d) shows similar trends with bias voltage as those extracted directly by PETR (Fig. S14(a)-(b). Hence, the results from both methods, as well as the spike segments detected by the PETR, are deemed reliable.

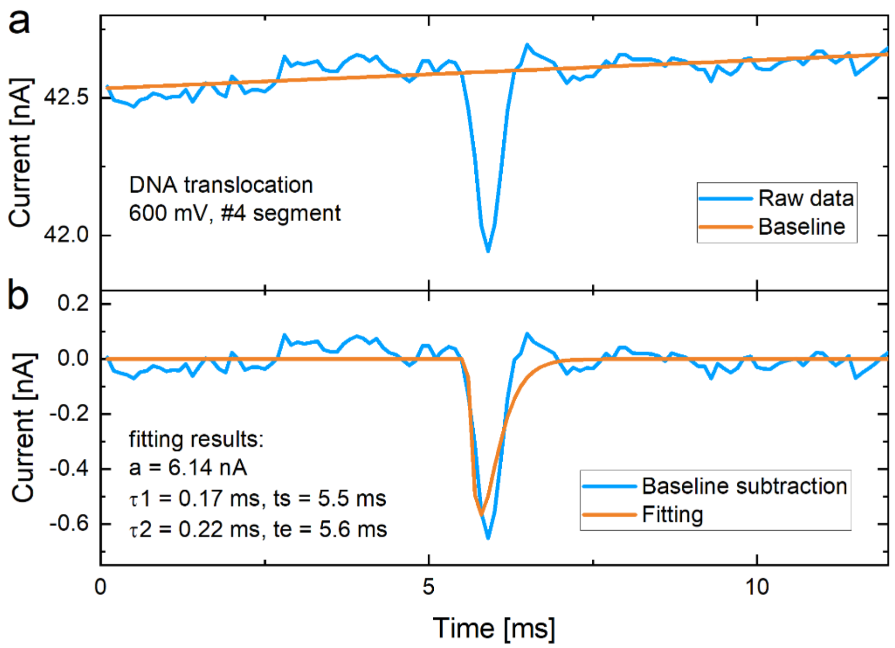

Fig. S16. Procedure of the ADEPT algorithm. (a) The raw spike segment and the baseline. (b) Fitting the spike by the target function on the baseline subtracted data.

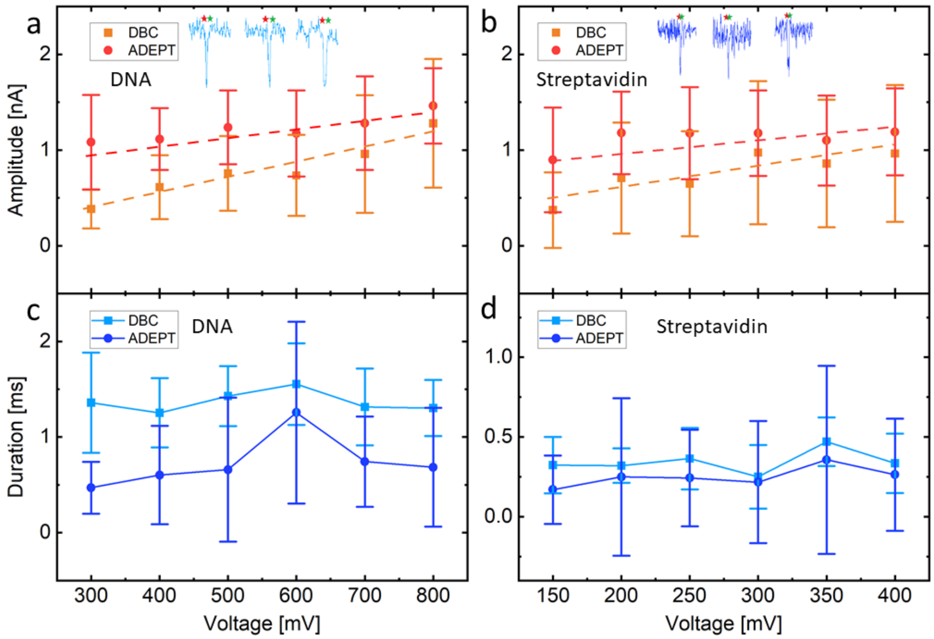

Fig. S17. Results from DBC and ADEPT as two demonstrations of further processing the output spike segments from PETR. Spike amplitude average with its spread of (a) DNA and (b) streptavidin translocation data. Spike duration average with its spread of (c) DNA and (d) streptavidin translocation data.

References:

(1) N. Carion, F. Massa, G. Synnaeve, N. Usunier, A. Kirillov, S. Zagoruyko, End-to-End Object Detection with Transformers. **2020**, 2005.12872, *arXiv*, https://arxiv.org/abs/2005.12872 (accessed April 17, 2022).

(2) R. Girshick, J. Donahue, T. Darrell, J. Malik, Rich Feature Hierarchies for Accurate Object Detection and Semantic Segmentation. **2014**, 1311.2524, *arXiv*, https://arxiv.org/abs/1311.2524 (accessed April 17, 2022).

(3) M. Tan, R. Pang, Q. V. Le, EfficientDet: Scalable and Efficient Object Detection. **2020**, 1911.09070, *arXiv*, https://arxiv.org/abs/1911.09070 (accessed April 17, 2022).

(4) J. Redmon, S. Divvala, R. Girshick, A. Farhadi, You Only Look Once: Unified, Real-Time Object Detection. **2016**, 1506.02640, *arXiv*, https://arxiv.org/abs/1506.02640 (accessed April 17, 2022).

(5) C. Wen, D. Dematties, S.-L. Zhang, A Guide to Signal Processing Algorithms for Nanopore Sensors, *ACS Sensors* **2021**, 6, 3536–3555.

(6) D. Dematties, C. Wen, M. D. Pérez, D. Zhou, S.-L. Zhang, Deep Learning of Nanopore Sensing Signals Using a Bi-Path Network. *ACS Nano* **2021**, 15, 14419−14429.

(7) S. Zeng, C. Wen, P. Solomon, S.-L. Zhang, Z. Zhang, Rectification of Protein Translocation in Truncated Pyramidal Nanopores, *Nat. Nanotechnol.* **2019**, 14, 1056–1062.

(8) Z. Gu, Y.-L. Ying, C. Cao, P. He, Y.-T. Long, Accurate Data Process for Nanopore Analysis, *Anal. Chem.* **2015**, 87, 907–913.

(9) A. Balijepalli, J. Ettedgui, A. T. Cornio, J. W. Robertson, K. P. Cheung, J. J. Kasianowicz, C. Vaz, Quantifying Short-Lived Events in Multistate Ionic Current Measurements, *ACS Nano* **2014**, 8, 1547–1553.
